# Intranasal administration of BReC-CoV-2 COVID-19 vaccine protects K18-hACE2 mice against lethal SARS-CoV-2 challenge

**DOI:** 10.1038/s41541-022-00451-7

**Published:** 2022-03-14

**Authors:** Ting Y. Wong, Katherine S. Lee, Brynnan P. Russ, Alexander M. Horspool, Jason Kang, Michael T. Winters, M. Allison Wolf, Nathaniel A. Rader, Olivia A. Miller, Morgane Shiflett, Jerilyn Izac, David Varisco, Emel Sen-Kilic, Casey Cunningham, Melissa Cooper, Holly A. Cyphert, Mariette Barbier, Ivan Martinez, Justin R. Bevere, Robert K. Ernst, F. Heath Damron

**Affiliations:** 1 Department of Microbiology, Immunology, and Cell Biology, West Virginia University, Morgantown, WV USA; 2 Vaccine Development Center at West Virginia University Health Sciences Center, Morgantown, WV USA; 3 Fina Biosolutions, LLC, Rockville, MD USA; 4 Department of Microbial Pathogenesis, University of Maryland School of Dentistry, Baltimore, MD USA; 5 Department of Biological Sciences, Marshall University, Huntington, WV USA; 6 West Virginia University Cancer Institute, Morgantown, WV USA; 7 School of Medicine, Morgantown, WV USA

**Keywords:** Protein vaccines, Conjugate vaccines, Adjuvants

## Abstract

SARS-CoV-2 is a viral respiratory pathogen responsible for the current global pandemic and the disease that causes COVID-19. All current WHO approved COVID-19 vaccines are administered through the muscular route. We have developed a prototype two-dose vaccine (BReC-CoV-2) by combining the Receptor Binding Domain (RBD) antigen, via conjugation to Diphtheria toxoid (EcoCRM^®^). The vaccine is adjuvanted with Bacterial Enzymatic Combinatorial Chemistry (BECC), BECC470. Intranasal (IN) administration of BreC-CoV-2 in K18-hACE2 mice induced a strong systemic and localized immune response in the respiratory tissues which provided protection against the Washington strain of SARS-CoV-2. Protection provided after IN administration of BReC-CoV-2 was associated with decreased viral RNA copies in the lung, robust RBD IgA titers in the lung and nasal wash, and induction of broadly neutralizing antibodies in the serum. We also observed that BReC-CoV-2 vaccination administered using an intramuscular (IM) prime and IN boost protected mice from a lethal challenge dose of the Delta variant of SARS-CoV-2. IN administration of BReC-CoV-2 provided better protection than IM only administration to mice against lethal challenge dose of SARS-CoV-2. These data suggest that the IN route of vaccination induces localized immune responses that can better protect against SARS-CoV-2 than the IM route in the upper respiratory tract.

## Introduction

As of January 2020, when the first SARS-CoV-2 genome was released, tremendous progress has been made in developing vaccines against COVID-19. To date, there are greater than 200 vaccines being developed worldwide to combat SARS-CoV-2, the causative agent of the COVID-19 pandemic^1^. Currently, there are eight vaccines that have been approved by WHO for administration that are being used around the world and more than 8 billion COVID-19 vaccines that have been given worldwide^2^. Approved vaccinations for COVID-19 and most vaccines in development have been administered or designed to be given through the intramuscular route. Few COVID-19 vaccines under development are administered through the nasal route. Each route of vaccination provides a unique protection profile for respiratory viruses. Intramuscular vaccination produces a predominantly systemic immune response dominated mostly by serum IgG and, resulting in minimal to no detectable mucosal immune response at the site of infection^3,4^. The vaccine response generated after intramuscular immunization can leave the upper respiratory tract vulnerable to viral replication and dissemination because it lacks the mucosal immune response generated by natural infection or intranasal vaccination^3^. However, intranasal vaccination may provide both a systemic and a robust local IgA response, as what occurs during natural infection, which may ultimately lead to total protection^3^. In pre-clinical studies, non-human primates vaccinated intramuscularly with Pfizer-BioNtech (BNT162b2) intramuscularly and then challenged with SARS-CoV-2 had detectable SARS-CoV-2 RNA copies in the nasal and oropharyngeal swabs collected after challenge^5^. We hypothesize that a vaccine must induce both mucosal and systemic immune responses to achieve sterilizing immunity against SARS-CoV-2.

Vaccine platforms that utilize nanoparticles, carrier proteins, and virus like particles (VLPs) can enhance the immunogenicity of antigens by increasing the size and quantity of the antigen presented to the immune system^6^. Novavax utilizes recombinant nanoparticle technology to increase immunogenicity of the spike protein in their COVID-19 vaccine formulation^7^. SpyBiotech and Serum Institute of India have developed a recombinant protein vaccine utilizing Hepatitis B surface antigen VLP to display RBD in order to strengthen immunogenicity of RBD^8^. In our studies, we have generated a recombinant COVID-19 vaccine containing the receptor binding domain (RBD) of the SARS-CoV-2 spike antigen conjugated to EcoCRM^®^ (an *E. coli* expressed CRM_197_)^9^. Crosslinking of CRM197 and a candidate target antigen protein can create nanoparticle-like structures containing multiple copies of the target antigen. CRM_197_ has been used in licensed vaccines for *Streptococcus pneumoniae, Haemophilus influenzae b*, and *Neisseria meningitidi*s to help increase the immunogenicity of polysaccharide antigens by promoting a T cell-dependent response^10–13^. CRM_197_ has also been used to enhance the immunogenicity of weakly immunogenic proteins, such as malaria proteins^14,15^.

Optimal COVID-19 vaccine immunity requires the activation of both cellular and humoral responses in regard to (1) activation of CD4 T cells to activate B-cell maturation to produce functional antibodies to neutralize SARS-COV-2 as well as B-memory responses and 2) stimulation of CD8 T cell production to eliminate virus-infected cells and the activation of CD8 T memory cells^16^. Vaccine adjuvants allow for the enhancement of both cellular and humoral immune responses that are necessary for COVID-19 vaccine immunity. Bacterial Enzymatic Combinatorial Chemistry (BECC) is a novel adjuvant methodology developed to synthesize TLR4-agonists, lipid A mimetics. The BECC system uses lipid A biosynthetic and/or modification enzymes expressed in a bacterial background to rationally engineer lipid A structures with altered binding to the host TLR4 receptor and immunostimulatory properties^17^. BECC adjuvants have been successfully used in both viral and bacterial pre-clinical vaccine formulations and are shown to generate a balanced Th1/Th2 response^18^. Viral vaccine studies with Influenza virus H1N1 showed decreased viral titers and weight loss when influenza hemagglutinin antigen was adjuvanted with BECC470, as well as elicited a balanced Th1/Th2 immune response^19^.

Overall, the aim of this study was to evaluate the efficacy of intramuscular and intranasal vaccination with BReC-CoV-2 against SARS-CoV-2 using the K18-hACE2 mouse challenge model^20–25^. We hypothesized that intranasal immunization, which induces a combination of both mucosal and systemic immune responses, would lead to better protection against SARS-CoV-2 than intramuscular vaccination with BReC-CoV-2. Here, we describe a series of murine immunogenicity and challenge studies that led us to a protective vaccine formulation and route of administration. Our findings demonstrate that, unlike intramuscular administration, intranasal administration of BReC-CoV-2 provided protection against lethal doses of both the ancestral strain as well as Delta SARS-CoV-2.

## Results

### Assessing different combinations of RBD-EcoCRM^®^ and adjuvants in vaccine formulations against SARS-CoV-2

**Fig. 1:**
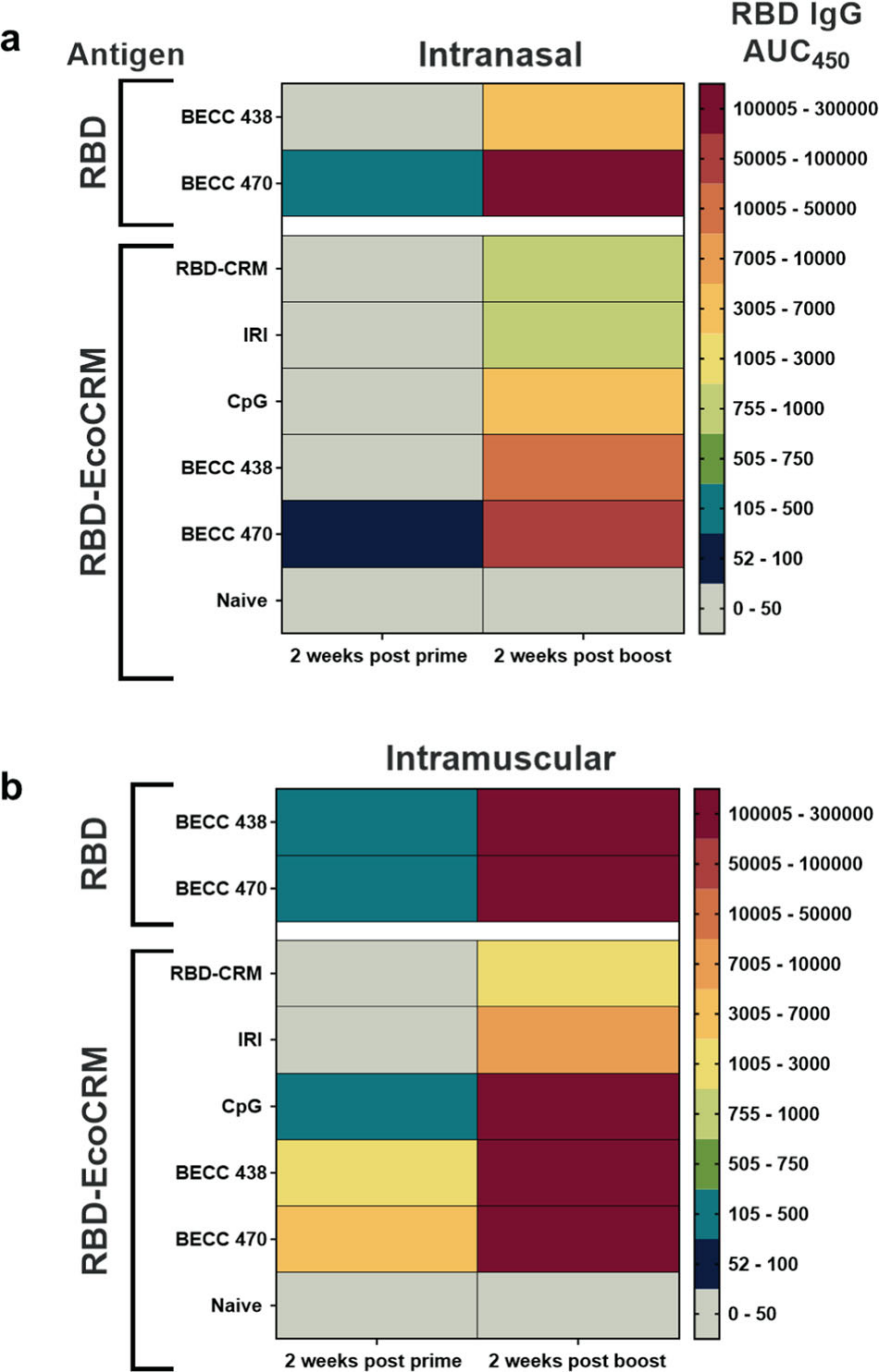
Mouse immunogenicity studies to identify vaccine candidates. 7 COVID-19 vaccine formulations were administered intranasally (**a**) or intramuscularly (**b**) in CD-1 mice in two doses. Heat map depicts the AUC450 values from RBD-IgG titers at 2 weeks post prime (left) and 2 weeks post boost (right). The maximum AUC450 value is set at 300,000, and the minimum is at 0.

It has been hypothesized that nasal vaccination offers a unique protection profile and advantages to muscular vaccination against SARS-CoV-2^3^. To test this hypothesis, we evaluated numerous antigens and adjuvants to identify a highly immunogenic vaccine formulation that would be subsequently evaluated in a K18-hACE2-transgeneic mouse model. Since RBD has a small molecular weight, to improve responses to RBD, we conjugated RBD to EcoCRM^®^, a genetically detoxified diphtheria toxoid carrier protein^26^. The conjugation of RBD to EcoCRM^®^ yielded a product with approximately one EcoCRM^®^ fused to 7–8 RBD molecules. The purpose behind the crosslinking of RBD with EcoCRM^®^ was to enhance the immunogenicity and subsequent recognition of RBD by the immune system. Based on vaccine immunogenicity screens in CD1 mice (Fig. 1), we hypothesized that a TLR4-agonist adjuvant would promote a robust antibody response. To test this hypothesis, we utilized Bacterial Enzymatic Combinatorial Chemistry (BECC). BECCs, are a TLR4-agonist that can help drive a balanced Th1/Th2 immune response that can help clear viral infections. We evaluated different adjuvants including: CpG (TLR9 agonist), IRI-1501 (Beta-glucan from yeast), BECC438 (biphosphorylated lipid A), and BECC470 (monophosphorylated lipid A) (Supplementary Table 1). In these studies, female CD1 outbred mice were immunized with the vaccine formulations indicated in Supplementary Table 1, either through an intranasal or intramuscular route. Mice were boosted 3 weeks later with the same formulation through the same routes. Serological analysis was performed at 2 weeks post prime and 2 weeks post boost (Fig. 1). Overall, mice demonstrated modest improvement in immunogenicity with RBD-EcoCRM^®^ compared to RBD alone supplemented with different adjuvant combinations, both through the IM and IN routes. Intramuscular administration of RBD or RBD-EcoCRM^®^ adjuvated with BECC438 resulted in similar RBD IgG titers at 2 weeks post boost; however, when administered intranasally, RBD-EcoCRM^®^ with BECC438 elicited greater RBD IgG titers compared to RBD (Fig. 1). Intranasally, BECC470 induced similar RBD-IgG responses formulated with RBD or RBD-EcoCRM (Fig. 1a). Intramuscular vaccination with RBD-EcoCRM^®^ adjuvanted with CpG generated increased RBD IgG titers compared to intranasal vaccination (Fig. 1a, b). In humans, CpG is only administered IM and would not likely be an ideal candidate IN adjuvant. RBD-EcoCRM^®^ adjuvanted with BECC470 generated a robust RBD-IgG response both intranasally and intramuscularly compared to other adjuvants tested (Fig. 1).

### RBD-EcoCRM^®^ adjuvanted with BECC470 elicits robust antibody responses in CD1 mice

**Fig. 2:**
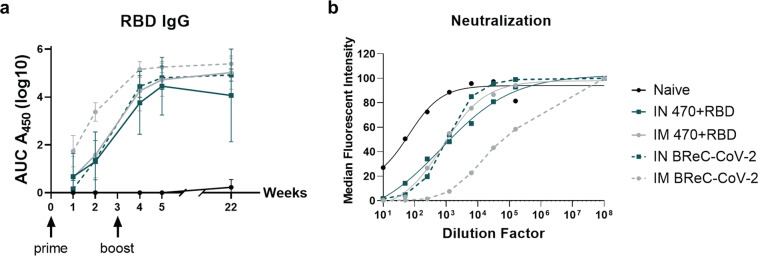
Analysis of antibody responses and neutralization capacity of IN and IM BreC-CoV-2 vaccines. CD-1 mice were IN or IM vaccinated with BECC470 with RBD or RBD- EcoCRM^®^ in two doses. **a** Serum was taken at 1 week and 2 weeks post prime, 1 week and 2 weeks post boost and 22 weeks post boost. Log10 AUC450 values from RBD-IgG titers are depicted for each vaccine. Results shown as mean ± SD. **b** In vitro neutralization assay performed on the Luminex platform. Serum was obtained from 2 weeks post boost. Naïve represents the group that received no vaccine, BReC-CoV-2 denotes mice immunized with RBD- EcoCRM^®^ adjuvanted with BECC 470, 470 represents BECC470 adjuvant, and receptor binding protein (RBD).

In this study, we focused on further investigating RBD-EcoCRM^®^ and BECC470 (BReC-CoV-2). Initially, IM BReC-CoV-2 generated elevated production of RBD IgG titers after 1 week and 2 weeks post prime compared to the other vaccines (Fig. 2a). We observed that IN and IM administration of BReC-CoV-2 produced robust RBD-IgG titers in the serum after boost (Fig. 2a). The IN BReC-CoV-2 generated a 3-log increase of anti-RBD IgG 1-week post boost from 2 weeks post prime. Whereas the IM BReC-CoV-2 produced a 2-log increase of anti-RBD IgG from 2 weeks post prime to 1-week post boost (Fig. 2a). Overall, at 1 and 2 weeks post prime IM BReC-CoV-2 generated significant anti-RBD IgG titers compared to IN BreC-CoV-2 vaccination (Supplementary data 1). However, there were no statistical differences measured between RBD alone and RBD-EcoCRM^®^ with BECC470 (Supplementary Data 1). An ideal COVID-19 vaccine would need to protect long-term; therefore, we measured RBD IgG titers at 22 weeks post boost were consistent with 2 weeks post boost in both IN and IM BReC-CoV-2 vaccinated groups (Fig. 2a). In addition to serological analyses, we also confirmed antibodies generated were able to neutralize RBD binding to ACE2 in vitro at 2 weeks post boost (Fig. 2b). Overall, the collective data from the pilot immunogenicity study indicated that IM and IN BReC-CoV-2 vaccines produced long-lasting strong anti-RBD IgG responses.

### Intranasal administration of BReC-CoV-2 protected mice from SARS-CoV2 challenge

**Fig. 3:**
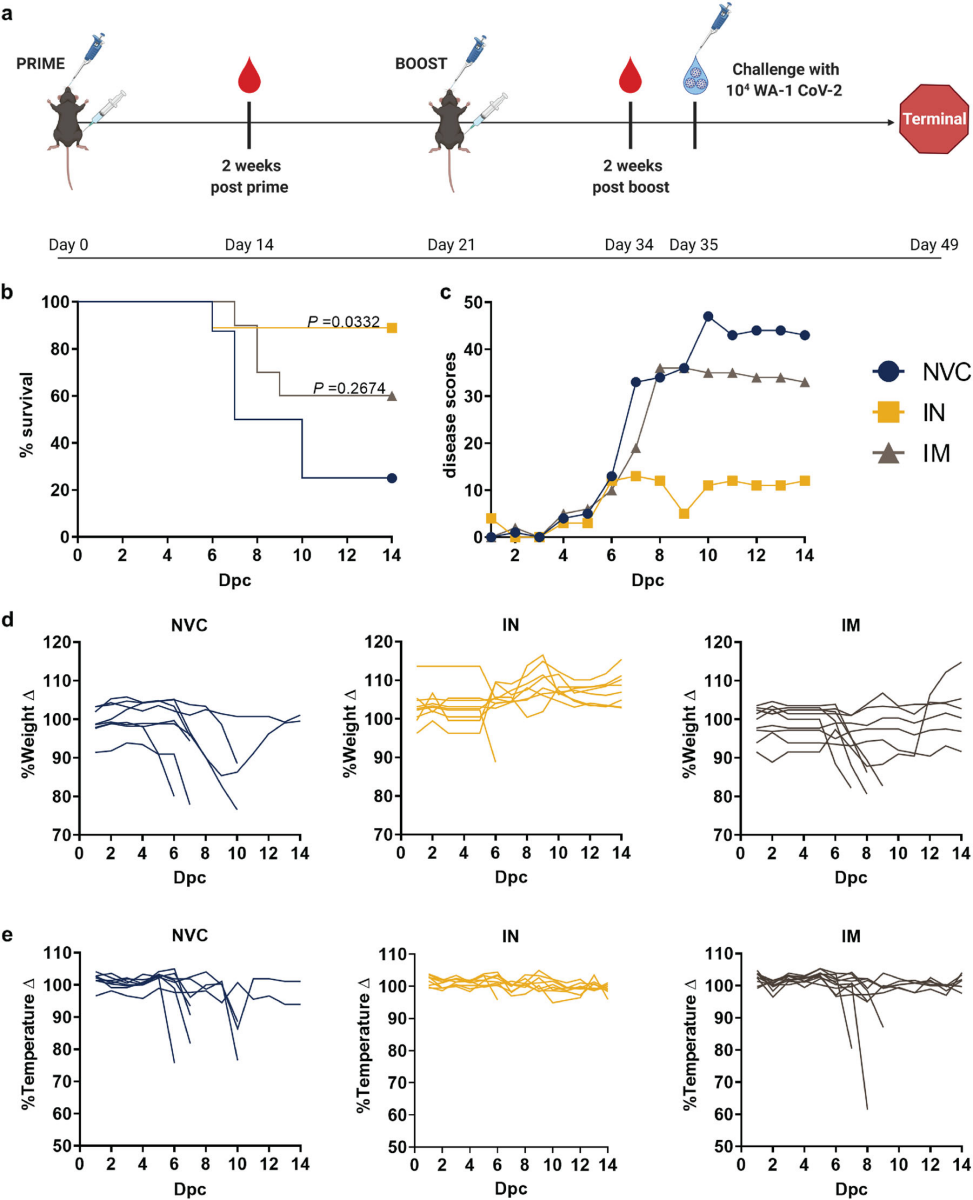
Intranasal administration of BReC-CoV-2 protected mice from SARS-CoV2 challenge. **a** vaccine and challenge schematic in K18-hACE2 mice. Mice were primed and boosted with either IN or IM BReC-CoV-2, and blood for serological analysis was collected 2 weeks post prime and boost. Mice were challenged intranasally with 10^4^ PFU/dose of WA-1 SARS-CoV-2, and mice were monitored for 14 days post challenge. **b** NVC (*n* = 8), IN BreC-CoV-2 (*n* = 9), and IM BreC-CoV-2 (*n* = 10) vaccinated animals Kaplan Meier survival curve. NVC had 25%, IN had 89%, and IM had 60% survival. Log-rank (Mantel–Cox) test were used to test significance of survival between sample groups. **c** Disease scores were calculated each day for each mouse and added per group. If a mouse reached a disease score of 5 or above, the mouse was euthanized, but the score was retained downstream for disease score analysis. **d** % weight change from 100% of the NVC, IN, and IM groups. **e** % temperature change from 100% of the NVC, IN, and IM groups.

We next tested the protective capacity of IN and IM BReC-CoV-2 in a SARS-CoV-2 challenge model. K18-hACE2 mice were vaccinated with IN and IM formulations of BReC-CoV-2 (Fig. 3a). At 2 weeks post boost, IN (*n* = 9), IM BReC-CoV-2 (*n* = 10), and no vaccine challenged (NVC) (*n* = 8) groups were challenged with a 10^4^ PFU/dose of WA-1 strain of SARS-CoV-2 and monitored for disease outcomes for a 14-day period. We assessed disease manifestations such as weight loss, appearance, activity, eye closure, respiration, and hypothermia (Supplementary Fig. 1). Mice were euthanized if they achieved a disease score 5 or greater, which determined that they were morbid. We calculated the cumulative disease score by adding the total scores of each mouse in one group. When animals became morbid and required euthanasia, we retained the score of the animal in the sum of the remaining days of the experiment. This disease scoring system helped us predict when mice would become morbid and is inverse to the falling Kaplan Meier curve. Throughout this 14-day period, we observed that NVC and IM BReC-CoV-2 vaccinated mice began decreasing in weight at day 7 post challenge, whereas the IN BReC-CoV-2 vaccinated mice gradually gained weight (Fig. 3d). IN vaccinated animals compared to NVC and IM maintained stable rectal temperatures throughout the 2-week monitoring period which corroborated their disease scores (Fig. 3c, e). However, NVC and IM vaccinated mice rectal temperature plummeted at days 7 and 8 post challenge (Fig. 3e). Unlike the IM, NVC was not able to recover in temperature as IM vaccinated mice. When evaluating the groups based on their disease scores, NVC began to increase in disease scores at day 7 and continually increased in disease scores until day 10 (Fig. 3c). IM BReC-CoV-2 vaccinated mice peaked in disease scores at day 8, but then returned to normal health scores throughout the rest of the challenge trial (Fig. 3c). IN BReC-CoV-2 vaccinated mice maintained low disease scores throughout the entirety of the 14-day monitoring period compared to both the NVC and IM vaccinated mice (Fig. 3c). Weight and temperature loss along with disease scores correlated with poor survival outcome (25% survival) in NVC group (Fig. 3b). IM vaccinated mice portrayed a better disease outcome than NVC, with 60% survival (Fig. 3b), and IN vaccinated mice experienced significant survival compared to NVC (*P* = 0.0332) with 89% survival (Fig. 3b). Overall, the protection profile indicated that IN vaccination with BReC-CoV-2 compared to IM and NVC protected mice from SARS-CoV-2 challenge suggesting that the mucosal immune response may play a role in driving protection from SARS-CoV2.

### IN vaccination with BReC-CoV-2 decreases viral RNA burden in the lung and brain

**Fig. 4:**
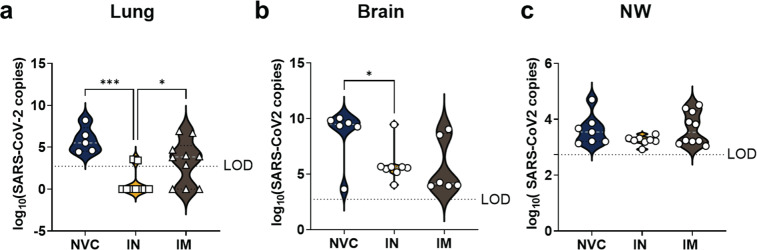
Determination of viral RNA levels in challenged mice. 100 ng of lung and brain homogenate was used to perform qPCR analysis on the SARS-CoV-2 nucleocapsid RNA. **a** Violin plots depicting the SARS-CoV-2 viral RNA copies in the right lobe of the lung, with white dotted line representing the median for each group plot. Ordinary one-way ANOVA with Sidak’s multiple comparisons test was used to perform statistical analysis. *P* = 0.0007***, and *P* = 0.0436*. **b** Violin plots depicting the SARS-CoV-2 viral RNA copies in the left lobe of the brain, with white dotted line representing the median for each group plot. Unpaired T-test was performed for statistical analysis. *P* = 0.0230*. **c** 500 µL of nasal wash (NW) was assessed for qPCR quantification of viral nucleocapsid RNA. Violin plots representing the SARS-CoV-2 viral RNA copies.

As the disease monitoring data suggested, IN vaccination with BReC-CoV-2 was superior in protection compared to IM mice. To corroborate the observed disease monitoring data, the viral RNA burden of the vaccinated mice compared to the NVC was determined. For this analysis, we measured RNA copies of nucleocapsid to SARS-CoV-2 in the lung (Fig. 4a), brain (Fig. 4b), and nasal wash (NW) for each animal (Fig. 4c). In the lung, IN vaccination of BReC-CoV-2 significantly decreased viral RNA compared to NVC and IM vaccinated BReC-CoV-2 (Fig. 4a) indicating that IN vaccination limits viral RNA burden. Studies have shown that K18-hACE2 mice succumb to SARS-CoV-2 brain infection after challenge^22,27,28^. IN vaccination with BreC-CoV-2 significantly decreased viral RNA in the brain compared to NVC suggesting that IN vaccination prevented the dissemination of virus into the brain (Fig. 4b). IN vaccination also decreased viral copies in the NW compared to NVC and IM; however, these differences were not statistically significant (Fig. 4c). Overall, there was a significant reduction of viral RNA copies in the lung of IN-vaccinated mice compared to IM and NVC, a significant decrease of viral RNA in the brain compared to NVC as well as fewer viral RNA copies in the NW. Decreased detection of viral RNA suggested that IN BReC-CoV-2 diminished viral replication at the site of infection aided in survival compared to IM BReC-CoV-2.

### Both IM and IN BReC-CoV-2 RBD antibody responses increase during SARS-CoV-2 challenge

**Fig. 5:**
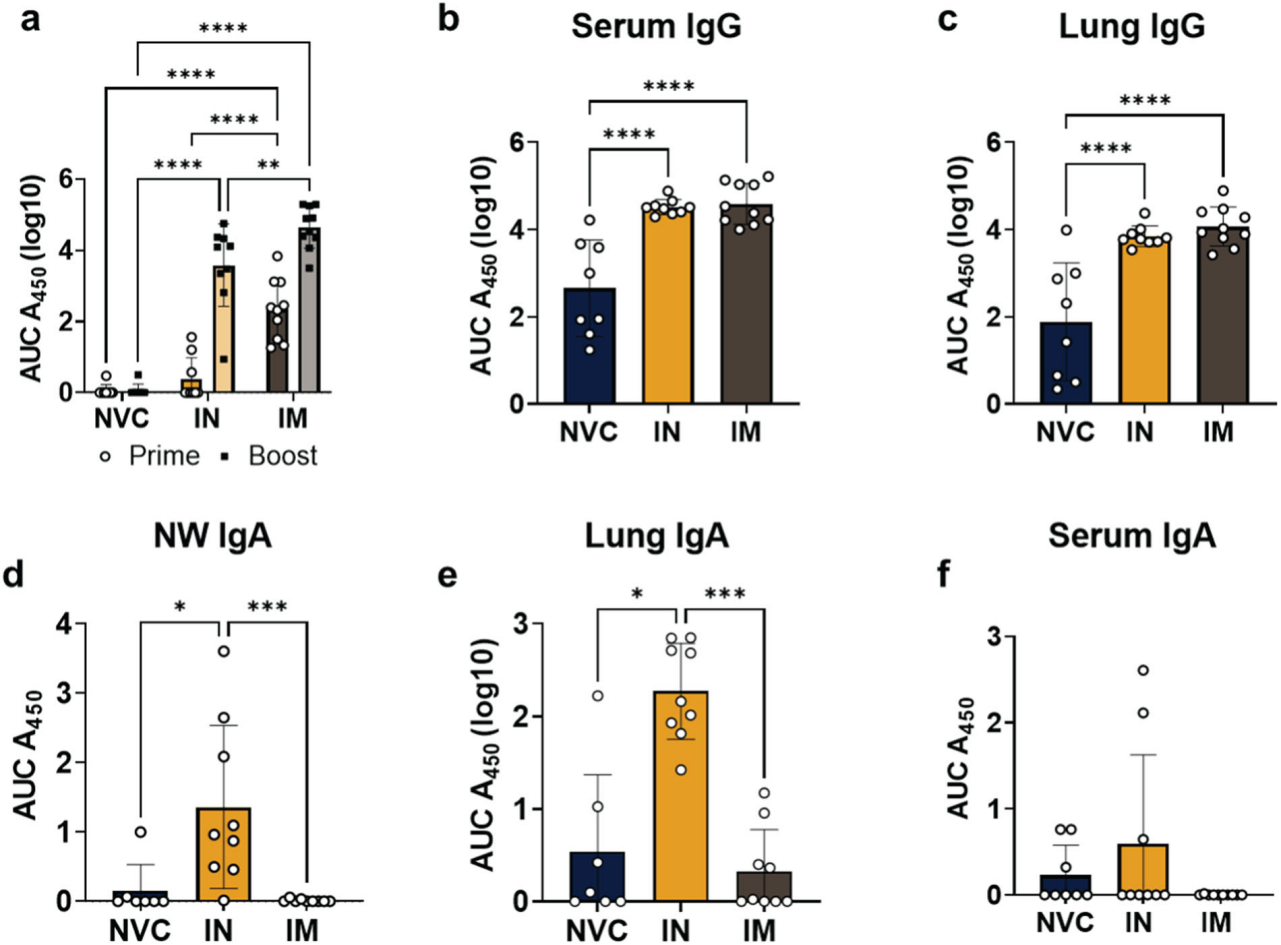
Serological analysis of serum, lung, and nasal antibodies. RBD IgG and IgA titers represented by log10 AUC450 values. Results represented as mean ± SD. **a** Pre-challenged NVC, IN and IM RBD IgG titers at 2 weeks post prime (left column, circles) and 2 weeks post boost (right column, squares). Two-way ANOVA with Tukey’s multiple comparisons test was used to determine *P* values. *p* < 0.0001****, *p* = 0.0051**. **b** Serum RBD-IgG titers post challenge. One way ANOVA performed for statistical analysis with Tukey’s multiple comparisons test. *p* < 0.0001****. **c** Lung supernatant RBD-IgG titers post challenge. One way ANOVA performed for statistical analysis with Tukey’s multiple comparisons test. *p* < 0.0001****. **d** NW RBD-IgA titers post challenge. Kruskal–Wallis test with Dunn’s multiple comparisons test performed for statistical analysis. *P* = 0.0009***, *p* < 0.0108*. **e** Lung supernatant RBD-IgG titers post challenge. Kruskal–Wallis test with Dunn’s multiple comparisons test performed for statistical analysis. *P* = 0.0009***, *P* = 0.0129*. **f** Serum RBD-IgA titers post challenge.

To investigate the antibody responses generated by BReC-CoV-2 vaccination, we first analyzed RBD-specific IgG production systemically and then locally in the lung. Systemic RBD IgG was measured before challenge and after challenge with SARS-CoV-2. In order to measure serum RBD IgG before challenge, blood was collected at 2 weeks post prime and 2 weeks post boost (Fig. 5a). At 2 weeks post prime, both IN and IM begin to generate detectable RBD IgG titers, with the IM generating higher RBD titers than both NVC and IN (Fig. 5a). Both IN and IM BReC-CoV-2 vaccinated groups induced a robust response to boosting, but IM vaccination elicited increased RBD-IgG titers compared to IN and NVC (Fig. 5a). Post challenge, serum RBD IgG was significantly elevated in both IN and IM vaccinated groups compared to NVC suggesting challenge may have increased antibody production (Fig. 5b). In the lung supernatant, similar to the serum, RBD IgG were significantly increased in both the IN and IM vaccinated mice compared to the NVC (Fig. 5c) indicating no difference between the IN and IM RBD IgG titers in the lung.

#### IN BReC-CoV-2 generated a robust localized IgA response compared to IM vaccination

To characterize the mucosal antibody response to BReC-CoV-2 vaccination, IgA titers were measured in the lung and nasal wash. In the lung supernatant, NVC and IM vaccinated mice did not generate RBD-specific IgA compared to IN BReC-CoV-2 vaccination (Fig. 5e). To further confirm the findings that the mucosal antibody response was contributing significantly to protection, anti-RBD IgA in the nasal wash was analyzed. Similar to the lung supernatant, IN vaccination significantly increased RBD-IgA compared to the undetectable IgA amounts in the NVC and IM vaccinated groups (Fig. 5d). Serum RBD IgA titers were also examined. The results indicated that IgA was released systemically because pre-challenge IgA was slightly elevated in IN-vaccinated mice but not in the NVC and IM groups. However, post challenge, there was no change in the serum RBD IgA titers in any groups (Fig. 5f). In summary, both IN and IM vaccination generated similar IgG responses in the lung and serum. However, IN vaccination induced a stronger IgA response in the lung and NW compared to IM, suggesting that the mucosal antibody response is potentially important in facilitating clearance of SARS-CoV-2 in the respiratory tract.

### BECC470 induces Th1/Th2 responses in both IN and IM BReC-CoV-2 vaccination

Previous pre-clinical vaccine studies using BECC470 as an adjuvant have shown that BECC470 generated a balanced Th1/Th2 immune response^19^. To investigate the Th1 and Th2 immune response elicited by IN and IM BReC-CoV-2 vaccination, IgG1 (Th2) and IgG2c (Th1) subtypes were analyzed in the serum. Both IN and IM BReC-CoV-2 vaccination induced significant RBD specific IgG2c and IgG1 responses compared to NVC (Supplementary Fig. 2). IM BReC-CoV-2 vaccination also generated a significant increase in IgG1 compared to IN vaccination indicating a Th2 biased response with IM vaccination compared to IN (Supplementary Fig. 2B). NVC mice had an expected increase in IgG2c compared to IgG1 indicating a Th1 response to viral infection (Supplementary Fig. 2). IgG2c/IgG1 ratios of less than one are considered Th1-biased whereas the ratio of greater than one would indicate Th2 responses. Overall, IN and IM vaccination-induced IgG1/IgG2c ratios of 0.7 and 0.9, respectively. Both vaccines induced Th2 responses, but IN immunization is driving slightly more Th1 antibody responses.

### Both IN and IM BReC-CoV-2 vaccination-induced neutralizing antibodies

**Fig. 6:**
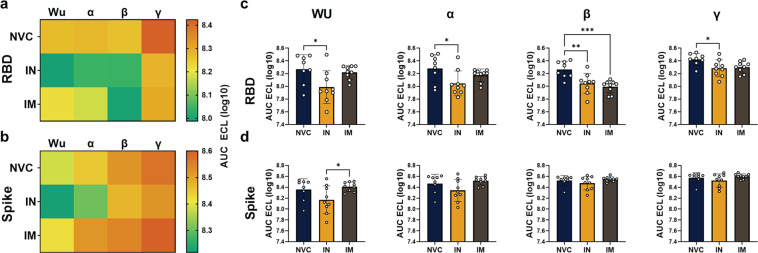
Analysis of RBD-ACE2 neutralization capacity of serum. MSD neutralization assay with RBD and Spike of the variants of concern with ACE2 was performed. All values are represented by the log10 AUC of the electrochemiluminescence emitted from the MSD plate reader. **a** Heat map depicts the neutralization capacity of challenged serum of NVC, IN, and IM groups against the RBD of different strains of SARS-CoV-2 (Wuhan, Alpha, Beta, and Gamma). **b** Heat map depicts the neutralization capacity of challenged serum of NVC, IN, and IM group against the Spike of different strains of SARS-CoV-2 (Wuhan, Alpha, Beta, and Gamma). **c**, **d** Individual values of the neutralization capacity of RBD from the heat map of RBD and spike represented by the log10 AUC of ECL. Results represented as mean ± SD. Two-way ANOVA with Tukey’s multiple comparisons test performed for statistical analysis. *P* = 0.0261* (RBD-Wu), *P* = 0.0322* (RBD-Alpha), *P* = 0.0062**, *P* = 0.0009*** (RBD-Beta) *P* = 0.0361* (RBD-Gamma), *P* = 0.0376* (Spike-Wu).

Antibody analysis of IN and IM BReC-CoV-2 vaccination detected high levels of RBD specific IgG and IgA; therefore, we determined if these antibodies were functional in neutralizing RBD binding to ACE2. The MSD COVID-19 ACE2 neutralization multiplex assay was used to analyze neutralization of the RBD and spike protein of the variants of concern (VOC) (Alpha, Beta, and Gamma). Neutralization of RBD or Spike binding to ACE2 was measured through electrical chemiluminescent (ECL) signal intensity for NVC, IN, and IM vaccinated mice. The higher the signal the less neutralization and the less intense the signal the more neutralization capability. Both IN and IM vaccinated mice had significant neutralizing antibody titers compared to NVC in the serum demonstrating that both IN and IM vaccination generated functional antibodies (Fig. 6). NVC mice, as expected, had no neutralization against the VOCs (Fig. 6). IN vaccinated mice had significantly higher neutralization capacity than NVC for Alpha, Beta, and Gamma (Fig. 6a, b); whereas IM vaccinated mice had increased neutralization capacity compared to NVC against Beta (Fig. 6c, d). For whole spike neutralization, IN vaccination generated significant neutralizing titers against the Wuhan strain of spike compared to IM vaccination (Fig. 6a, b). Overall, IN vaccination with BReC-CoV-2 showed superior neutralization capacity over IM in the ability to neutralize multiple VOCs RBD from binding to ACE2.

### Increased levels of serum CXCL13 in NVC and IM BReC-CoV-2 vaccinated mice indicate poor disease prognosis

**Fig. 7:**
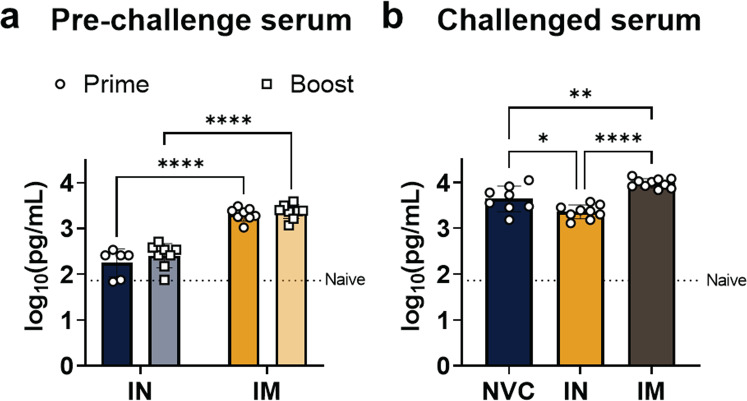
Analysis of CXCL13 in serum in relation to immunization. **a** CXCL13 (log10 pg/mL) in pre-challenged serum. Two-way ANOVA with Sidak’s multiple comparisons test was performed for statistical analysis. *P* < 0.0001****. Naïve baseline represented as dotted line at 1.861183. **b** Post challenged CXCL13 levels in the serum. Ordinary one-way ANOVA with Tukey’s multiple comparisons test was performed for statistical analysis. *P* = 0.0112*, *P* = 0.0018**, and *P* < 0.0001****. All results represented as mean ± SD.

CXCL13 is an important chemokine marker for germinal center activity, B-cell maturation, memory B-cell, and plasma cell formation. Conversely, in non-vaccinated COVID-19 patients, increased CXCL13 levels have been shown to be a marker of a poor clinical outcome compared to patients who survived COVID-19^29,30^. In the context of immunization (pre-challenge), CXCL13 was detectable in IN immunized mice, but higher in IM immunized mice, suggesting germinal centers were more active after IM immunization (Fig. 7a). After challenge, NVC mice had higher CXCL13 compared to naïve mice as would be expected (Fig. 7b). IN immunized mice had the lowest CXCL13 levels. These data suggest that germinal centers were not activated due to the mucosal protection and levels of circulating systemic antibodies in the IN immunized mice.

### IN BReC-CoV-2 decreased IFN-γ in the lung

SARS-CoV-2 is known to cause inflammation in the lung and induce interferon responses^22,23,31^. Therefore, we hypothesized that IN vaccination should help decrease inflammatory markers in the lung. To test this hypothesis, we measured inflammatory cytokines in the lung supernatant post SARS-CoV-2 challenge. Compared to the NVC and IM vaccination, IN vaccination significantly lowered IFNγ in the lung supernatant (Supplementary Fig. 3C), whereas other pro-inflammatory cytokines remained similar between NVC, IN, and IM (Supplementary Fig. 3a–d). C reactive protein (CRP) was also measured as a marker to evaluate inflammation during SARS-CoV-2 challenge. CRP was significantly decreased in IN and IM vaccinated groups compared to NVC in the lung (Supplementary Fig. 3D). Overall, vaccination decreased inflammation in the lung, with IN vaccination decreasing both IFN-γ and CRP compared to NVC.

### IM vaccination decreases both chronic and acute inflammation in the lung whereas IN vaccination decreases acute inflammation only

**Fig. 8:**
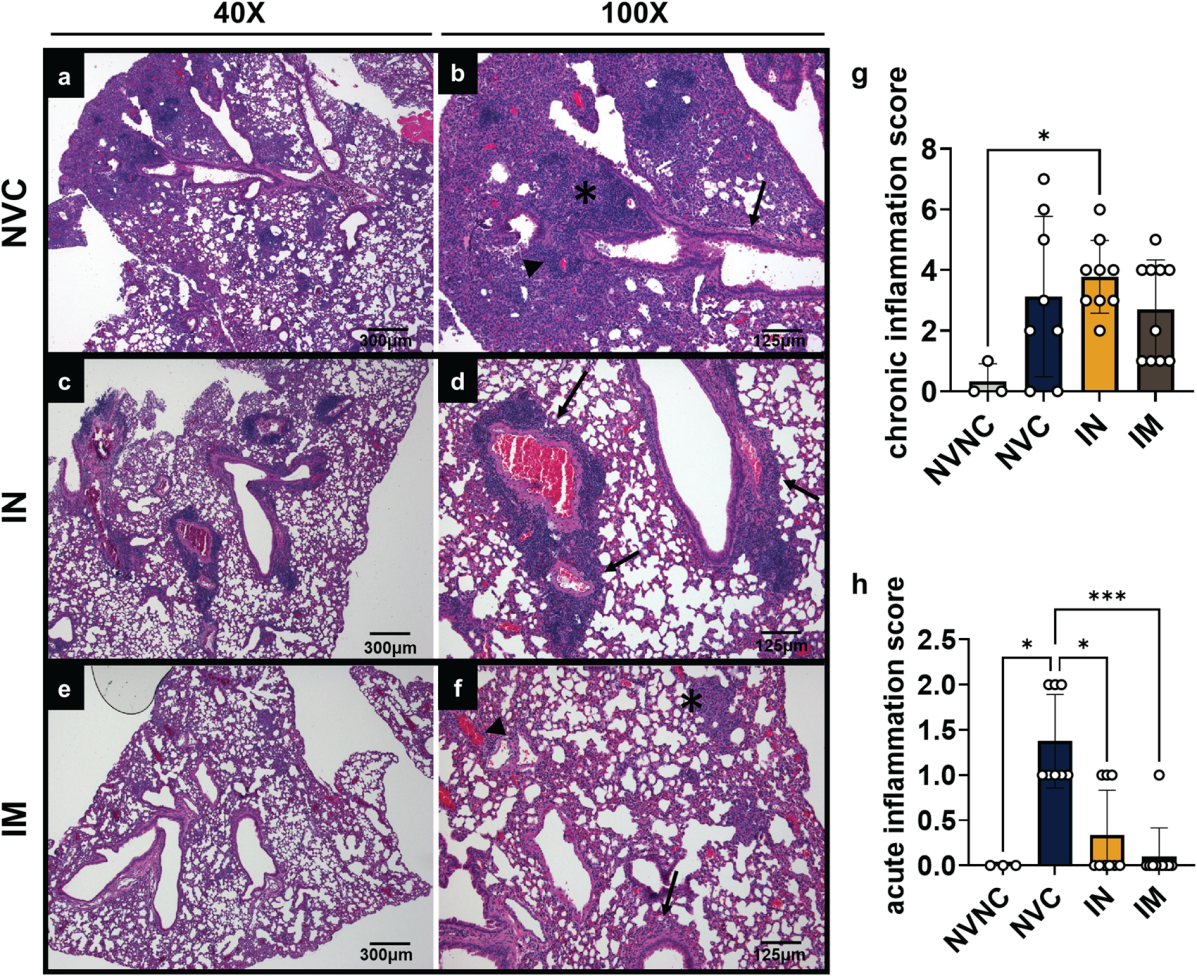
Histopathological analysis of naïve or vaccinated mice challenged with SARS-CoV-2. **a** 40× magnification of the lung of NVC (scale bar = 300 μm). **b** 100× magnification of 8A. Inflammation in the parenchyma is denoted by the asterisk, inflammation surrounding the blood vessel is marked by an arrowhead, and inflammation in the airways are denoted by an arrow (scale bar = 125 μm). **c** 40× magnification of the lung of the IN BReC-CoV-2 vaccinated representative mouse (scale bar = 300 μm). **d** 100× magnification of 8C. Arrows show inflammation in the airways (scale bar = 125 μm). **e** 40× magnification of the lung of the IM BReC-CoV-2 vaccinated representative mouse (scale bar = 300μm). **f** inflammation in the parenchyma is denoted by the asterisk, surrounding the blood vessels marked by an arrowhead and inflammation in the airways represented by arrows (scale bar = 125 μm). **g** Total chronic inflammation scores of each mouse. **h** Total acute inflammation score of each mouse. Results represented as mean ± SD. All statistical analysis was performed using Kruskal–Wallis test with Dunn’s multiple comparisons test. *P* = 0.0449* (chronic); *P* = 0.0143, 0.150*, 0.0004*** (acute).

We next hypothesized that IN vaccination would reduce total inflammation due to decreasing inflammatory cytokines in the lung. The left lobe of the lung was subjected to H&E staining and evaluated for histopathological analysis for chronic and acute inflammation. Chronic inflammation was scored by the presence of recruited lymphocytes, plasma cells, and macrophages in the parenchyma and blood vessels. Acute inflammation was denoted by the infiltration of neutrophils and the presence of edema in the parenchyma, blood vessels, and airways. IN vaccinated mice had increased chronic inflammation scores (3.8) compared to NVNC (0.33), NVC (3.1), and IM (2.7) with the presence of plasma cells, lymphocytes, and macrophages localized around blood vessels (Fig. 8c, d, h). IN mice scored an average inflammation score of 4.1, lower than NVC (Fig. 8g, h). Mice vaccinated IM with BReC-CoV-2 had the lowest chronic and acute inflammation scores compared to NVNC, NVC, and IN mice with an overall mean inflammation score of 2.8 (Fig. 8e–h). IM mice had mostly chronic inflammation found in the parenchyma, blood vessels, and bronchi (Fig. 8e–g). Overall, IM vaccinated mice had less acute and chronic inflammation than NVNC, NVC, and IN suggesting that IN vaccination mimicked natural infection by recruiting cells into the lung to fight viral infection.

### IN BReC-CoV-2 vaccination upregulates specific immune genes in response to SARS-CoV-2 challenge

**Fig. 9:**
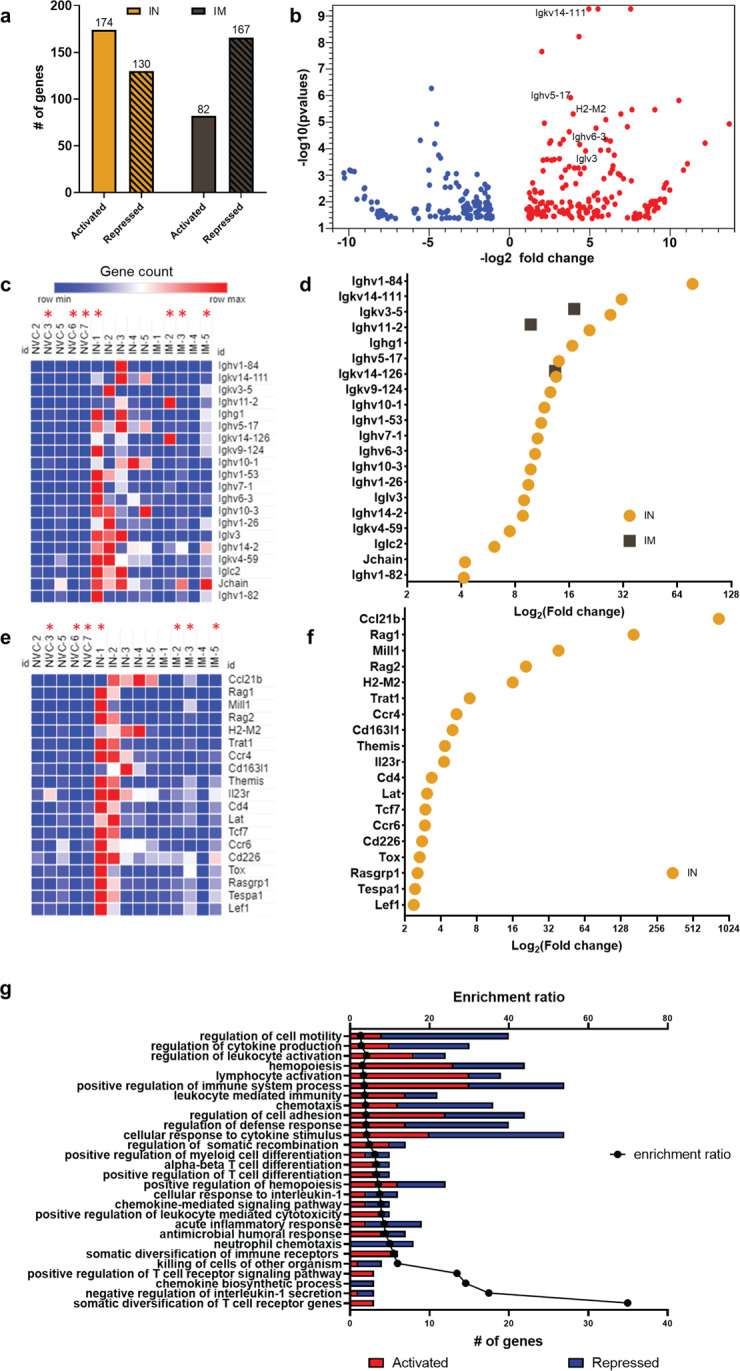
RNAseq analysis reveals IN BReC-CoV-2 vaccination results in unique gene expression signatures enriched for T cell responses. All analyses were performed on CLC genomics workbench 21. **a** Number of significant (FDR *p* < 0.05) activated and repressed genes in IN BReC-CoV-2 and IM BReC-CoV-2 groups. **b** Volcano plot indicating significant gene expression profile of IN BReC-CoV-2 compared to NVC. Red circles denote upregulated genes and blue circles represent downregulated genes. **c** Heat maps were generated by Morpheus. Heat map represents gene counts of immunoglobulin genes in each mouse lung. **d** Significant fold changes of the immunoglobulin genes of interests in both IN and IM BReC-CoV-2 groups. **e** Heat maps were generated by Morpheus. Heat map represents gene counts of adaptive immune response genes of interest in the mouse lung. **f** Significant fold changes of adaptive immune response genes in both IN and IM groups. IM BReC-CoV-2 did not have significant fold changes. Red asterisks next to the sample ID indicate mouse morbidity before the termination of the study. NVC3 and IN1 euthanized on day 6, IM5 euthanized on day 7, IM3 euthanized on day 8, IM2 euthanized on day 9, and NVC6 and 7 euthanized on day 10. **g** Gene set enrichment analysis of IN BReC-CoV-2 compared to NVC was performed on WEB-based Gene SeT AnaLysis Toolkit. The enrichment ratio of significant GO-terms compared to the number of genes in each enriched gene set. Red represents activated genes and blue represents repressed genes.

To capture the transcriptional profile of intranasal and intramuscular BReC-Cov-2 vaccination during SARS-CoV-2 challenge, the lung was analyzed using RNA sequencing. IN BReC-CoV-2 compared to NVC had 174 activated genes and 130 repressed genes whereas IM BReC-CoV-2 compared to NVC had 82 activated genes with 167 repressed genes (Fig. 9a and Supplementary Data 2). Immunoglobulin genes involved in regulating the adaptive immune response were significantly upregulated in IN BReC-CoV-2 vaccination and challenge compared to NVC (Fig. 9b–d). Genes responsible for general T-cell regulation and activation such as *Lat*, *Lef1*, *Mill1*, *Trat1*, *Tespa1*, *Themis*, *Tox*, *Tcf7, H2M2*, *Cd163l1*, *Cd226*, and *Cd4* hint at the presence of effector and resident T-cells in the lung (Fig. 9e–f) However, IM vaccinated compared to NVC only had three immunoglobulin genes (*Igkv3-5*, *Ighv11-2*, and *Igkv14-126)* significantly upregulated, and no significant fold changes in the adaptive immune response gene set (Fig. 9c, d). Over-Representation Analysis was used to enrich GO-terms of the biological processes in IN BReC-CoV-2 challenged mice compared to NVC. We observed gene set enrichment and significant upregulation in genes involved in a variety of important immune responses such as leukocyte activation, lymphocyte activation, leukocyte mediated immunity, somatic recombination, somatic diversification of immune receptors, and somatic diversification of T-cell receptor genes (Fig. 9g). Conversely, there were increased repressed genes involved in cellular response to interleukin-1 suggesting that IN BReC-CoV-2 helped decrease inflammation in the lung (Fig. 9g). Overall, the transcriptomic data generated from sequencing the lung from IN and IM BReC-CoV-2 vaccination during SARS-CoV-2 challenge mirrored the correlates of protection collected throughout this study.

### IM BReC-CoV-2 prime followed by IN boost afforded survival against SARS-CoV-2 Delta variant challenge

**Fig. 10:**
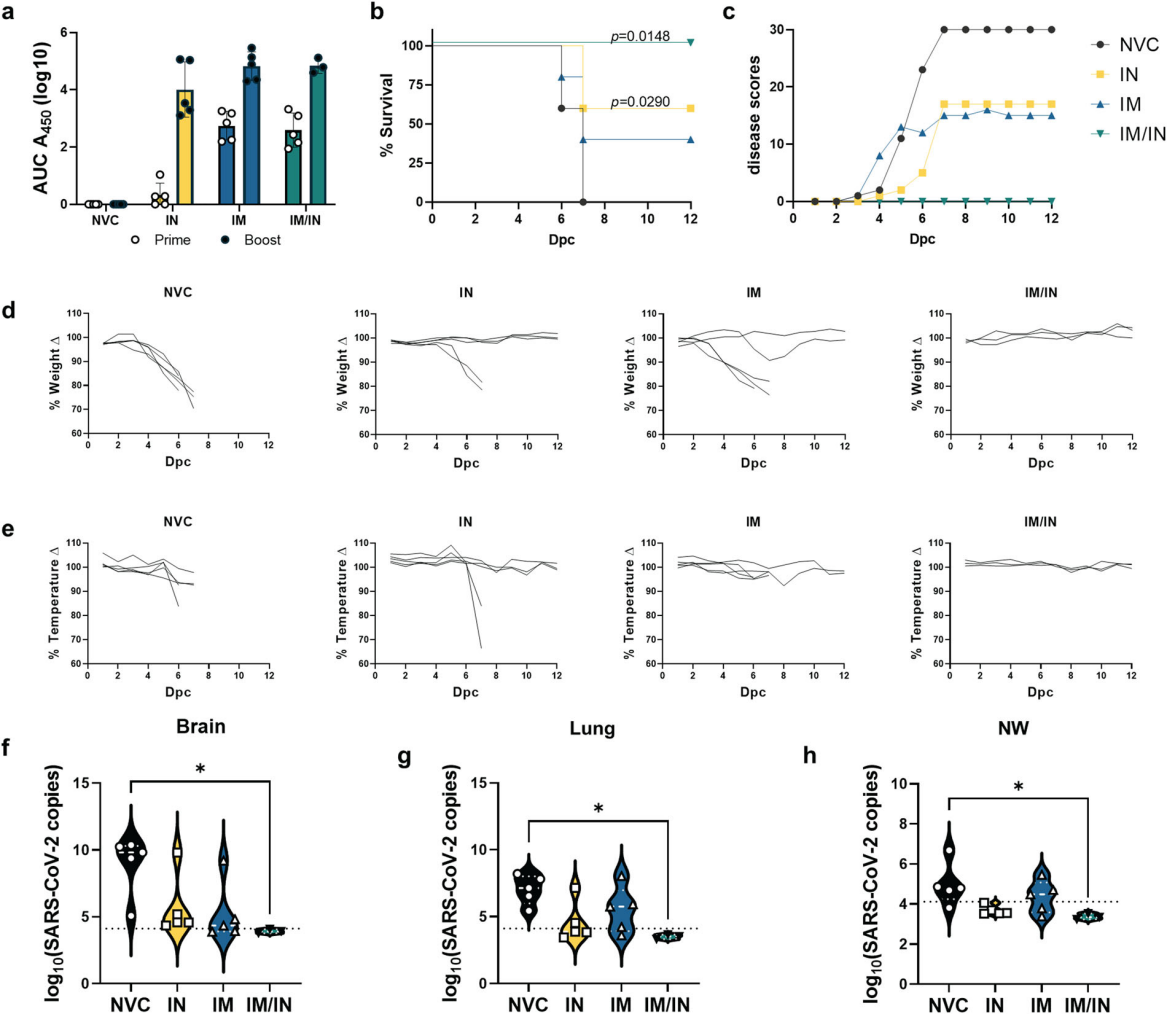
IM BReC-CoV-2 prime followed by IN boost afforded protection against SARS-CoV-2 Delta variant challenge. **a** Serological analysis of 2 weeks post prime and boost of RBD IgG titers. Boost time points were significant compared to prime. RBD IgG titers represented by log10 AUC450 values. **b** Kaplan– Meier survival curve of BReC-CoV-2 vaccinated mice. Mantel–Cox test used to calculate significance between IN, IM, and IM/IN BReC-CoV-2 compared to NVC. **c** Cumulative disease scores of NVC, IN, IM, and IM/IN throughout 12-day course of study. **d** % Weight change of NVC, IN, IM, and IM/IN BReC-CoV-2. **e** % temperature change of NVC, IN, IM, and IM/IN BReC-CoV-2. **f** Violin plots depicting the SARS-CoV-2 viral RNA copies in the brain, with white dotted line representing the median for each group plot. **g** Violin plots depicting the SARS-CoV-2 viral RNA copies in the right lobe of the lung, with white dotted line representing the median for each group plot. **h** 500 µL of nasal wash (NW) was assessed for qPCR quantification of viral nucleocapsid RNA. Violin plots representing the SARS-CoV-2 viral RNA copies. Ordinary One-way ANOVA with Tukey’s multiple comparisons test was performed on the brain (*P* = 0.0236*), lung (*P* = 0.0144*) and NW (*P* = 0.0391*).

SARS-CoV-2 Delta variant is the predominant circulating variant in the world as of December 2021^32–34^. Therefore, we wanted to evaluate whether BReC-CoV-2 vaccination can protect against Delta challenge in mice. K18-hACE2 mice were vaccinated with 2 doses of BReC-CoV-2 through the IN route, IM route and lastly, primed through the IM route and boosted through the IN route (IM/IN). IN and IM BReC-CoV-2 vaccination generated similar RBD IgG titers as the previous vaccine and challenge study with WA-1 (Fig. 10a). IM/IN vaccination elicited similar RBD IgG titers as IM vaccination (Fig. 10a). NVC (*n* = 5), IN (*n* = 5), IM (*n* = 5), and IM/IN (*n* = 3) were challenged with a lethal 10^4^ PFU/dose of Delta variant and monitored similarly for disease manifestations as the previous challenge trial with WA-1 SARS-CoV-2. NVC mice began succumbing to disease at day 6, and by day 7 post challenge, the remaining mice were morbid and were euthanized. The severity of disease caused by the Delta variant in the NVC group was reflected by the increase of the cumulative disease scores as well as in the sharp decrease in weight and temperature. (Fig. 10b–e). IN BReC-CoV-2 vaccinated mice had increased survival compared to NVC (60% survival); however, 2 mice succumbed to Delta at day 7 post challenge (Fig. 10b). Cumulative disease scores peaked at day 7 in the IN-vaccinated group mirroring the moribund mice. The morbid mice in the IN-vaccinated group had increased disease scores as well as decreased temperature and weight compared to the rest of the group (Fig. 10c–e). Mice administered BReC-CoV-2 through the IM route had increased mortality compared to IN with a 40% survival rate. Disease scores reflected the morbidity of the IM vaccinated mice; however, interestingly, IM mice that succumbed to disease had a sharp decrease in weight but maintained temperature unlike NVC and IN moribund mice (Fig. 10c–e). Remarkably, all mice vaccinated with BReC-CoV-2 through IM prime and IN boost strategy survived a lethal challenge against the Delta variant (Fig. 10b). IM/IN group maintained stable weight and temperature throughout the course of challenge, as well as did not exhibit disease manifestations observed in NVC, IN and IM groups (Fig. 10c–e). Viral RNA burden in the brain (Fig. 10f), lung (Fig. 10g), and NW (Fig. 10h) followed similar trends as the disease assessment and survival in IN, IM, and IM/IN BReC-CoV-2 immunized mice. Interestingly, despite IN BReC-CoV-2 having a better survival outcome than IM BReC-CoV-2 vaccinated mice, NVC, IN and IM groups had similar levels of viral RNA in the brain and lung (Fig. 10f, g). However, IN BReC-CoV-2 vaccinated mice had decreased viral burden in the NW compared to NVC and IM BReC-CoV-2 (Fig. 10h). The heterologous prime (IM) and boost (IN) strategy provided significant decrease of viral RNA in the brain, lung, and NW compared to NVC (Fig. 10f–h) suggesting that IM prime with BReC-CoV-2 followed by IN boost prevented viral dissemination. Overall, 10^4^ PFU/dose of SARS-CoV-2 Delta variant was a lethal dose in non-vaccinated mice. IM/IN BReC-CoV-2 vaccinated offered superior protection against lethal Delta challenge compared to NVC, IN, and IM vaccination. IN BReC-CoV-2 provided significant protection against Delta challenge compared to NVC; however, did not offer complete protection, and IM BReC-CoV-2 supplied limited protection against Delta.

## Discussion

For protection against respiratory pathogens, nasal vaccines can offer both localized protection at the site of infection and activate systemic responses. Very few nasal vaccines have been approved for human use. To the best of our knowledge, only two examples are on the market: FluMist^®^, a live attenuated influenza FDA approved vaccine for seasonal flu and Nasovac^®^, an H1N1 pandemic flu vaccine^35^. The chimpanzee adenovirus vectored vaccine encoding a pre-fusion stabilized spike (S) protein (ChAD-SARS-CoV-2-S) is an example of an adenovirus vectored COVID-19 vaccine that has been shown protective as a single dose nasal vaccination in non-human primates and other models as well^36,37^. AdCovid™ developed by Altimmune, is another adenovirus vectored (replication-deficient adenovirus type 5) intranasal vaccine expressing RBD instead of the spike protein. In pre-clinical studies, a single dose of AdCovid™ offered sterilizing immunity against SARS-CoV-2 challenge and induced a robust mucosal response in the respiratory tract in mice^38,39^. However, AdCovid™ demonstrated a lack of efficacy in phase 1 clinical trials and was discontinued. A few pre-clinical trials evaluating intranasal vaccines utilizing a recombinant spike protein with stimulator of interferon genes (STING) adjuvant showed robust systemic and localized immunogenicity^40^. And lastly, a live attenuated and vectored Newcastle Disease virus expressing spike protein demonstrated sterilizing immunity against SARS-CoV-2 when administered IN^41^. Collectively, these studies hint that IN vaccines can protect against SARS-CoV-2.

In this study, our objective was to develop and evaluate a nasal vaccine against SARS-CoV-2 using RBD conjugated to EcoCRM^®^ adjuvanted with BECC470 (BReC-CoV-2). Before we tested BreC-CoV-2 in a SARS-CoV-2 challenge model, we performed an intensive immunogenicity screen for immunogenic vaccine antigen and adjuvant combinations in outbred mice (Fig. 1). We screened 14 different vaccine combinations through both the intranasal and intramuscular route. Using the K18 hACE2 mouse model, we demonstrated that intranasal vaccination with BReC-CoV-2 offered protection against WA-1 SARS-CoV-2 compared to IM vaccination. We observed that IN vaccination with BReC-CoV-2 increased percent survival, decreased disease scores, and maintained weight and temperature in the IN group throughout infection compared to IM and NVC (Fig. 3). Intranasal vaccination was performed with 50 µL of vaccine in order to deposit vaccine in both the upper respiratory tract and lungs. It is likely that this would cause both mucosal and systemic immune responses. Nasal vaccination decreased viral burden in the lung compared to IM and NVC (Fig. 4), as well as increased RBD IgA titers in the lung and nasal wash compared to IM and NVC (Fig. 5). Increased neutralizing antibodies against RBD of the variants of concern (Alpha, Beta, and Gamma) were found with IN compared to IM and NVC (Fig. 6). Intranasal vaccination with BReC-CoV-2 decreased IFN-γ in the lung compared to IM and NVC (Supplementary Fig. 3). However, histopathological analyses showed an increase of the recruitment of lymphocytes, macrophages, and plasma cells to blood vessels in the lung compared to IM vaccination (Fig. 8). RNAseq analysis performed on the lungs demonstrated that IN BReC-CoV-2 vaccination upregulated more genes involved in the adaptive immune response compared to NVC and IM groups (Fig. 9). Since the Delta variant of SARS-CoV-2 was the predominant strain in the world at the time of this study, a Delta challenge was performed in BReC-CoV-2 vaccinated mice. Compared to the WA-1 challenge, IN BReC-CoV-2 had significant survival compared to NVC and decreased disease scores. However, heterologous prime boost of BReC-CoV-2 offered 100% survival against Delta challenge (Fig. 10).

In our BReC-CoV-2 formulation, we utilized a carrier protein and an adjuvant synthesized from bacterial components. Bacterial components can serve as potent adjuvants for either bacterial or viral vaccines. We used BECC470 as the candidate adjuvant to supplement RBD-EcoCRM. Compared to GSK MPLA, they are both engineered forms of lipid A, TLR4-agonists, and drive a robust Th1 immune response, but there are significant differences in the way that they are synthesized. BECC was developed as an alternative route to produce lipid A mimetics. It uses novel methodology that generates products that are cost effective and easy to produce^17^. Carrier proteins are another important vaccine component for small molecular weight antigens such as RBD to increase antigen presentation thus immunogenicity. EcoCRM^®^ the carrier protein of our immunogen is a genetically detoxified diphtheria toxoid originally expressed in *Corynebacterium diphtheriae*^9^. We used EcoCRM^®^ which is CRM_197_ expressed as a soluble, properly folded protein in the cytoplasm of an *E. coli* strain engineered to have an oxidative cytoplasm^26,42^. Crosslinking of a carrier protein and RBD, forming a high MW nanoparticle like construct capable of presenting multiple molecules of RBD are likely critical for the enhanced response to the conjugate versus RBD alone.

In our studies, we acknowledge that the K18-hACE2 mouse model contains limitations such as increased sensitivity to SARS-CoV-2 challenge because of elevated expression of human ACE2 in the mouse compared to humans such as in the brain^25^. The severity of this transgenic challenge model does likely have a caveat because brain SARS-CoV-2 infection is atypical of human infection^28,43^. Future studies are needed to evaluate IN and IM administration of BReC-CoV-2 in other rodent models such as the Syrian hamster model. The hamster model results in pneumonia^44^. Hamster ACE2 are similar to human ACE2 and disease phenotypes of SARS-CoV-2 infection recapitulate those of human pneumonia and inflammation in the hamster model^45^. Unlike the K18-hACE2 mouse model, hamsters do not succumb to brain encephalitis, the majority of the virus remains in the lungs, and may spread to the GI tract^25^.

Since the K18-hACE2 mouse model is sensitive to SARS-CoV-2, it was important to determine an appropriate lethal challenge dose to effectively evaluate vaccine protection. In previous studies, we evaluated 10^4^ (*n* = 12) and 10^5^ (*n* = 13) PFU/dose of SARS-CoV-2 WA-1 in K18-hACE2 mice^46^. We observed that 10^4^ and 10^5^ PFU/dose resulted in 11% survival and 0% survival, respectively. Our BReC-CoV-2 challenge study showed that 10^4^ PFU/dose of WA-1 resulted in 25% survival in the non-vaccinated, challenged mice similar to the preliminary dose study. Other studies have shown that approximately 10^4^ PFU/dose also show similar lethality in K18-hACE2 mice and 10^5^ PFU/dose results in 100% lethality^22,23,28^. Since the WA-1 viral stock that was used to challenge mice in this experiment was sequenced and contained no deletions in the furin cleavage site, discrepancies in mouse survival in the 10^4^-challenge dose could be due to deviations in delivery of the challenge dose per mouse. To further investigate the optimal dose for maximizing vaccine efficacy, more studies should be done characterizing the lethal and sublethal doses of SARS-CoV-2, especially in relation to VOC strains.

In our first protection study, we challenged mice with the ancestral SARS-CoV-2 WA-1; however, this clade of strain is currently virtually non-existent. Nevertheless, we evaluated the neutralizing capacity of sera of BReC-Cov-2 vaccinated mice to RBD and spike proteins from the VOCs (Fig. 6). Sera from mice IN immunized with BReC-CoV-2 vaccination were able to significantly inhibit hACE2 binding of the VOC RBDs. This suggests that IN administration of BReC-CoV-2 may be able to protect mice challenged with these VOCs. Since the Delta variant is currently the predominant global variant; we challenged BReC-CoV-2 vaccinated mice with Delta (Fig. 10). Even though, IN BReC-CoV-2 significantly improve survival compared to NVC, survival rate decreased from 89% with WA-1 challenge to 60% with Delta challenge, indicating a decrease in vaccine efficacy against the VOC. However, we demonstrated that mice immunized through the IM/IN vaccine strategy with BReC-CoV-2 had 100% survival against lethal Delta challenge suggesting that the IM/IN vaccine route is the optimal vaccine strategy with BReC-CoV-2 in this model. The RBD used in BReC-CoV-2 was generated from the WA-1 strain of SARS-CoV-2. Therefore, mutations in RBD will decrease antibody binding and virus neutralization which is likely causing decreased vaccine efficacy of IN and IM BReC-CoV-2. Our data suggest that administering a booster dose through the IN route after an IM prime might provide increased protection against SARS-CoV-2. Further investigation is needed to evaluate the correlates of protection of BReC-CoV-2 IM/IN compared to IN or IM only routes with Delta challenge.

Neutralizing antibodies are important in diminishing the replication of SARS-CoV-2, whereas CD4+ and CD8+ T-cells play a large role in clearing and controlling SARS-CoV-2 infection^16,47,48^. Studies have shown that in humans, resident T-cells in the lung instead of in circulation were linked with better disease prognosis and survival^49^. We appreciate that in other intranasal vaccination studies for bacterial and viral pathogens that T resident memory cells are elevated in the lung and nasal associated lymphoid tissue^50^. We hypothesize that since BECC470 is a driver of Th1 immune responses (Supplementary Fig. 2A) that IN BReC-CoV-2 will also elicit robust T resident memory responses that will contribute to protection. However, further investigation is needed to study T resident memory cells in the lung as well as the nasal associated lymphoid tissue in the mouse.

Next generation sequencing is a powerful platform that can be used to profile vaccine responses. In this study we used bulk RNAseq to characterize the transcriptomic landscape of BReC-CoV-2 vaccinated lungs against WA-1 SARS-CoV-2 challenge (Fig. 9). Interestingly, IN BReC-CoV-2 vaccinated and SARS-CoV-2 challenged lungs revealed activation of immunoglobulin genes compared to NVC suggesting the presence of antibody-producing B cells in the lungs which could have contributed to protection of the IN BReC-CoV-2 mice. These data corroborate with serological analysis of IN BReC-CoV-2 lung, where we observed the increased induction of RBD IgG and IgA titers. Human COVID-19 studies observe the presence of memory B cells in the lung 6 months post infection which hints at the importance of memory B cells for protection against SARS-CoV-2 infection^51^. Additionally, IN BReC-CoV-2 lung showed transcriptional signatures of genes involved in T-cell signaling and differentiation, suggesting the presence of CD4^+^ and CD8^+^ T cells as well as T resident memory cells, which also has been shown in human COVID-19 cases^51^. *Rag1* and *rag2* were significantly upregulated in IN BReC-CoV-2 vaccination suggesting that mature B and T cells were residing in the lung. Remarkably, we only observed differentiation in the immune response genes in IN BReC-CoV-2 lungs and not in the IM BReC-CoV-2 lungs hinting that a localized immune response was occurring in the IN vaccinated. Overall, traditional RNAseq provides a snapshot of the immune response occurring during IN and IM BReC-CoV-2 vaccination; however, it does not detail antigen specificity of the immunoglobulin genes expressed (Fig. 9). Novel technology such as linking B cell receptor to antigen specificity through sequencing can aid in discovering antigen-specific B and T cell receptors that are crucial to a protective vaccine response.

In summary, our study demonstrates that intranasal administration of BReC-CoV-2 confers protection against WA-1 SARS-CoV-2 challenge in hACE2 mice compared to intramuscular vaccination. IN administration with BReC-CoV-2 protected transgenic mice against challenge, but also reduced viral burden in the lung, inhibited hACE2 binding of VOC RBDs, and induced high titers of IgA in the lung and nasal wash. Importantly, we also demonstrated that BReC-CoV-2 administered via an IM prime and IN boost strategy protected transgenic mice from a lethal challenge of the Delta variant. In the future, our goal is to evaluate BReC-CoV-2 in the Syrian hamster model with emerging VOCs such as the Delta variant. We also want to further investigate the mucosal IgA response of nasal BReC-CoV-2 in the lungs and nasal tissue. In summary, intranasal vaccination with BReC-CoV-2 offered better protection at the site of infection than intramuscular vaccination, indicating that intranasal route of this vaccine candidate can be pursued in future studies.

## Methods

### Animal welfare, biosafety, and ethics statements

CD1 outbred mouse immunogenicity studies were performed under the approved West Virginia University IACUC protocol number 2004034204 whereas B6.Cg-Tg(K18-ACE2)2Prlmn/J mouse vaccine and SARS-CoV-2 challenge studies were executed under IACUC protocol number 2009036460. All mice were humanely euthanized based on the disease scoring system, described below (Supplementary Fig. 1), and no deaths occurred in the cage. All SARS-CoV-2 challenge studies were conducted in the West Virginia University Biosafety Laboratory Level 3 facility under the IBC protocol number 20-04-01. SARS-CoV-2 samples were inactivated with 1% Triton per volume or Trizol before exiting high containment.

### Mouse vaccination

Female outbred CD1 mice were obtained from Charles River Laboratories (strain code: 022) at 4 weeks old and vaccinated at 8 weeks of age. Both male and female B6.Cg-Tg(K18-ACE2)2Prlmn/J mice were purchased from Jackson Laboratory (stock no: 034860) at 8 weeks old and vaccinated at 10 weeks old for the WA-1 challenge study. Female B6.Cg-Tg(K18-ACE2)2Prlmn/J mice were vaccinated at 13 weeks old and were used in the Delta (B.1.617.2) challenge study. Both CD1 and K18-hACE2 mice were administered 50 μL immunizations through either the intramuscular route or intranasal route. For intranasal immunization, mice were anesthetized through intraperitoneal injection with ketamine/xylazine per approved protocols, then administered 25 μL of vaccine into each nare.

### Production of antigen

RBD of the Wuhan original strain of SARS-CoV-2 was recombinantly produced by transient transfection in HEK293T cells using a pCAGGS expression vector with RBD construct with a C-terminal hexahistidine tag and codon optimized for mammalian expression (pCAGGS vector catalog #: NR-52309 BEI Resources)^9^. RBD was then chemically conjugated to the carrier protein EcoCRM^®^ by Fina Biosolutions LLC (Rockville, MD).

### Determination of RBD-CRM ratio by mass spectrometry

Proteins RBD, CRM, RBD-CRM (1 µg each) were electrophoresed in SDS-PAGE gel. The protein bands were excised and extracted protein was treated with trypsin. The resulting peptides were analyzed on a Q Exactive™ Plus Hybrid Quadrupole-Orbitrap™ Mass Spectrometer and peptide spectra matched (PSM) were aligned to RBD or CRM proteins. Unique peptides were determined and the RBD to CRM ratio was determined. CRM and RBD individual resulted in 150 PSM or 16.2 per pmol of protein, respectively. Conjugated RBD-CRM resulted in 112 PSM (0.74pmol) of CRM and 95 PSM (5.86 pmol) of RBD. 5.86/0.74 pmol results in a ratio of 7.92 RBD per CRM of conjugated antigen.

### Vaccine composition

20 μg of RBD- EcoCRM^®^ was used in the vaccine formulations. The adjuvants BECC 470 and BECC 438 were obtained from Dr. Robert Ernst at the University of Maryland^17^. Briefly, 50 μg BECC 470 or BECC 438 were sonicated in a water bath sonicator for 15 min prior mixing with RBD-EcoCRM^®^ for 2 h before vaccination. IRI-1501 beta glucan was provided by Immunoresearch. CpG adjuvant was acquired from Dynavax.

### Luminex Magpix platform in vitro neutralization assay

Neutralization assay was developed using the Luminex Magpix platform^29^. Briefly, 1:2 dilution of mouse serum was added to Greiner black non-binding 96 well plates. Serum was diluted 1:5 down the plate. Luminex Magpix^®^ Microspheres (MC10012-YY) conjugated to RBD were added to the serum dilutions. After a 2 h incubation period, plates were washed 2× with 1× PBS-TBN on a 96 well magnet, ACE2-biotin was added to the plates and incubated for 1 h. Plates were washed again 2× on the magnet, and Streptavidin-phycoerythrin was added to the plates and incubated for 30 min at room temperature at 700 rpm. After the Streptavidin-phycoerythrin incubation, plates were washed again, and 100 μL of 1×PBS-TBN was added to plates and analyzed on the Magpix to measure neutralizing ability of serum antibodies.

### Serological analysis

ELISAs were performed to assess the total IgG (Novus Biologicals NBP1-75130), and IgA (Novus Biologicals NB7504) in the serum, lung supernatant, and nasal wash^29,46^. Total IgG titers were quantified in the serum and lung. High binding plates (Pierce 15041) were coated overnight at 4 °C with 2 μg/mL of RBD in phosphate-buffered saline. Plates were then blocked with 3% non-fat milk in PBS-0.1% Tween 20 overnight in the 4 °C. After blocking, 1:20 dilution of serum/lung supernatant from mice was added in the first row and diluted 1:2 down two plates (15 dilutions total) in 1% non-fat milk in PBS-0.1% Tween 20 leaving the last row on the last plate as a blank. Plates were incubated for 10 min at room temperature with shaking. Plates were then washed with PBS-0.1%Tween20 4 times, then goat-anti-mouse secondary IgG HRP (1:2000 dilution) was added to the plates and incubated as above (Novus Biosolutions). ELISAs were developed using TMB reagent (Biolegend 421101) (1:1 ratio) in the dark for 10 min, and the reaction was stopped using 25 μL 2N sulfuric acid. ELISAs were read using the Synergy H1 plate reader at 450 nm. Nasal wash, serum, and lung supernatant IgA titer quantification was performed using the same coating and blocking procedures as mentioned above. In separate ELISA assays, 100 μL of nasal wash, 1:20 dilution of serum, and 1:5 dilution of lung supernatant was added to the first rows of high binding plates and diluted down 2 plates at 1:2 dilution in 1% non-fat milk in PBS-0.1% Tween 20. Serum, nasal wash and lung supernatant samples were incubated for 2 h at room temperature with shaking. Plates were washed according to the protocol mentioned above. Secondary goat-anti-mouse IgA HRP (1:10,000) (Novus biologicals) was used in these assays and incubated for 1 h at room temperature with shaking. IgA ELISAs were developed with TMB substrate (1:1) for 20 min in the dark before adding stopping solution and read on the Synergy H1 plate reader at 450 nm. Serological data was also analyzed as antibody titer, IC50, and AUC. From our analysis, data followed nearly identical trends of titers per vaccine/control group, as well as have the same statistical significance (one-way ANVOVA using Tukey’s multiple comparisons test) between each method analyzed. Titers were represented as Area Under the Curve values calculated via GraphPad Prism v9.0.0.

### SARS-CoV-2 propagation and mouse challenge

SARS-CoV-2 USA-WA-1/2020 (NR-52281) (GenBank accession number: MN985325) or SARS-CoV-2 Delta variant B.1.617.2 hCoV-19/USA/WV-WVU-WV118685/2021 (GISAID Accession ID: EPI_ISL_1742834) were the challenge strains used in K18-hACE2 vaccine studies. SARS-CoV-2 USA-WA-1/2020 (NR-52281) was obtained from BEI and hCoV-19/USA/WV-WVU-WV118685/2021 was obtained from at patient sample at WVU. Both strains were propagated in Vero E6 cells (ATCC-CRL-1586) and re-sequenced. K18-hACE2 mice were challenged with a 10^4^ PFU/dose. Viral dose was prepared from the first passage of WA-1 at a concentration of 3.7 × 10^6^ PFU/mL diluted to a working concentration of 10^6^ PFU/mL. B.1.617.2 10^4^ PFU/dose was prepared from the first passage of a viral stock concentration of 8.25 × 10^5^ PFU/mL. Briefly, mice were anesthetized with IP injection of ketamine (Patterson Veterinary 07-803-6637)/xylazine (Patterson Veterinary 07-808-1947), and a total of 50 μL of 10^4^ PFU SARS-CoV-2 WA-1 or Delta was administered intranasally (25 μL per nare).

### Disease score of SARS-CoV-2 challenged mice

Challenged K18-hACE2 mice were evaluated daily through both in-person health assessments in the BSL3 and SwifTAG Systems video monitoring for 12–14 days. Health assessments of the mice were scored based on five criteria: (1) weight loss (scale 0–5), (2) appearance (scale 0–2), (3) activity (scale 0–3), (4) eye closure (scale 0–2), and (5) respiration (scale 0–2) (Supplementary Fig. 1). All five criteria were scored based off a scaling system where 0 represents no symptoms and the highest number on the scale denotes the most severe phenotype^52^. Weight loss(0–5) was scored based off percent weight loss from original weight before challenge using the scale 0–5%(0), 5–10%(1), 10–15%(2) 15–20%(3), > 20%(4, 5). If mice reached 20% weight loss before the termination of the study, mice were humanely euthanized at that time point. Appearance(0–2) was scored by observation of piloerection of fur, score of(0) indicative of groomed, healthy fur whereas score of (2) represented ungroomed fur. Activity(0–3) was scored based off(0) normal activity for the time of day observed and (3) collapsed or immobile. Eye closure(0–2) was assigned(0) for mice with open eyes and (2) mice with eye discharge in both eyes in addition to eye closure. Lastly, respiration was scored visually(0) mice with 80–200 breaths per minute and (2) irregular breathing, or gasping marked by fewer than 80 or more rapid than 200 breaths per minute. Additive disease scores of the five criteria were assigned to each mouse after evaluation. Mice that scored an additive disease score of 5 or above among all five criteria, weight loss of 20% or greater during the health assessment, or a respiration score of 2 required immediate euthanasia. Cumulative disease scoring was calculated by adding the disease scores of each mouse from the group. Morbid mice that were euthanized during the study, before day 14, retained their disease score for the remainder of the experiment.

### Euthanasia and tissue collection

Challenged mice that were assigned a health score of 5 or above or reached the end of the experiment were euthanized with an IP injection of Euthasol (390 mg/kg) (Pentobarbital) followed by a secondary measure of euthanasia with cardiac puncture. Blood from cardiac puncture was collected in BD Microtainer gold serum separator tubes, centrifuged at 15,000 × *g* for 5 min and serum collected for downstream analysis. Nasal wash was acquired by pushing 1 mL of PBS through the nasal pharynx. 500 μL of nasal wash was added to 167 μL of TRI reagent for RNA purification and the remainder of the nasal wash was frozen for serological analysis. Lungs were separated into right and left lobes. Right lobe of the lung was homogenized in 1 mL of PBS in gentleMACS C tubes (order number: 130-096-334) using the m_lung_02 program on the gentleMACS Dissociator. 300 μL of lung homogenate was added to 167 μL of TRI Reagent (Zymo research) for downstream RNA purification and 300 μL of lung homogenate was centrifuged at 15,000 × *g* for 5 min and the lung supernatant was collected for downstream analyses. The brain was excised from the skull and separated into the right and left hemispheres. The right hemisphere was homogenized in 1 mL PBS in gentleMACS C tubes using the same setting as lung on the gentleMACS Dissociator. 167 μL of TRI Reagent was added to 500 μL of brain homogenate for RNA purification.

### qPCR SARS-CoV-2 viral copy number analysis of lung, brain, and nasal wash

RNA purification of the lung, brain, and nasal wash was performed using the Direct-zol RNA miniprep kit (Zymo Research R2053) following the manufacturer’s protocol. SARS-CoV-2 copy numbers were assessed through qPCR using the Applied Biosystems TaqMan RNA to CT One Step Kit (Ref: 4392938). We utilized nucleocapsid primers (F: ATGCTGCAATCGTGCTACAA; R: GACTGCCGCCTCTGCTC); and TaqMan probe (IDT:/56-FAM/TCAAGGAAC/ZEN/AACATTGCCAA/3IABkFQ/) that were synthesized according to Winkler et al., 2020^23^. The following final concentrations were used according to the Applied Biosystems TaqMan RNA to CT One Step Kit manufacturer protocol: TaqMan RT-PCR Mix 2X, Forward and reverse primers 900 nM final, TaqMan probe 250 nM final, TaqMan RT enzyme mix 40X, and RNA template 100 ng (with the exception of nasal wash). Nasal wash RNA concentrations were not quantifiable on the Qubit 3 fluorometer; therefore, we used 5.4 μL of nasal wash RNA per reaction instead of 100 ng. Triplicates were prepared for each sample, and samples were loaded into a MicroAmp Fast optical 96 well reaction plate (Applied Biosystems 4306737). Prepared reactions were run on the StepOnePlus Real-Time System machine using the parameters: Reverse transcription for 15 min at 48 °C, activation of AmpliTaq Gold DNA polymerase for 10 min at 95 °C, and 50 cycles of denaturing for 15 s at 95 °C and annealing at 60 °C for 1 min.

### Meso scale discovery cOVID-19 ACE2 neutralization assay

SARS-CoV-2 challenged serum was analyzed using the SARS-CoV-2 Plate 7 Multi-Spot 96-well, 10 spot plate following the manufacturer protocol (catalog #: N05428A-1) on the MSD QuickPlex SQ120. The 10 spots contained: (1) CoV-2 Spike (2) RBD B.1.351 (3) CoV-2 N (4) RBD P.1 (5) BSA (6) RBD B.1.1.7 (7) Spike P.1 (8) Spike B.1.1.7 (9) Spike B.1.351 and (10) CoV2 S1 RBD. Three dilutions of serum, 1:5, 1:50, and 1:500 were analyzed on the MSD neutralization assay for each mouse to perform Area Under the Curve analysis on the electrochemiluminescence using GraphPad Prism.

### Cytokine analysis

R&D 5-plex mouse magnetic Luminex assay (Ref LXSAMSM) was used to quantify cytokines: CXCL13, TNFα, IL-6, IFN-γ, and C reactive protein in the serum and lung supernatant. Manufacturer protocols were followed in preparing samples. 5 plex mouse cytokine plate was analyzed on the Luminex Magpix and pg/mL were calculated based off standard curves generated for each cytokine in the assay.

### Histopathology

Left lobes of lungs from each mouse in the NVC, IN and IM groups in the WA-1 challenge study were fixed in 10 mL of 10% neutral buffered formalin. Fixed lungs were paraffin-embedded into 5 μm sections. Sections were stained with hematoxylin and eosin and sent to iHisto for pathological analysis. The pathologist was blinded to the experimental groups but was aware of groups that were challenged or not challenged with SARS-CoV-2. Lung samples were scored for chronic and acute inflammation in the lung parenchyma, blood vessels, and airways. Each mouse was scored individually using a standard qualitative toxicologic scoring criteria: 0-none; 1-minimal; 2-mild; 3-moderate; 4-marked; 5-severe. Chronic inflammation was denoted by the presence of lymphocytes and plasma cells and acute inflammation was scored by the presence of neutrophils and edema.

### Illumina library preparation, sequencing, and bioinformatic analysis

RNA quantity was measured with Qubit 3.0 Fluormeter using the RNA high sensitivity (Life Technologies) and RNA integrity was assessed on an Agilent Bioanalyzer 2100 Eukaryote Total RNA Nano chip (Applied Biosystems). RNA was DNAased before library preparation. Illumina sequencing libraries were prepared with KAPA RNA HyperPrep Kit with RiboErase (Basel, Switzerland). Resulting libraries passed standard Illumina quality control PCR and were sequenced on an Illumina NovaSeq s4 4000 at Admera Health (South Plainfield, NJ). A total of 100 million 2 × 150 bp reads were acquired per sample. Sequencing data will be deposited to the Sequence Read Archive. The reads were trimmed for quality and mapped to the *Mus musculus* reference genome using CLC Genomics Version 21.0.5. An exported gene expression browser table is provided as supplemental materials Supplementary data 2. Statistical analysis was performed with the Differential Gene Expression tool and genes were annotated with the reference mouse gene ontology terms. Genes with an FDR *p* value of <0.05 were considered differentially regulated. Volcano plot was generated with statistically significant genes. Genes of interest were plotted in a heat map that was generated in GraphPad version 9.0. Genes that were differentially regulated were further analyzed via the online WEB-based GEne SeT AnaLysis Toolkit using over-representation analysis using the mouse enrichment category gene ontology and biological process. Heat maps were generated using Morpheus^53^.

### IgG1/IgG2c subtypes

ELISAs were performed on the challenged serum to assay IgG1 (Novus Biologicals NB7511) and IgG2c (Novus Biologicals NBP2-68519) titers. ELISAs were coated with RBD following the same concentration and procedures mentioned above. Plates were blocked with 3% non-fat milk in PBS-0.1% Tween 20 for one hour at room temperature with shaking at 480 rpm. Serum concentration (1:20) was used as above following a 10 min incubation period. Secondary IgG1-HRP and IgG2c-HRP were used at a 1:10,000 dilution in 1% non-fat milk in PBS-0.1% Tween 20 with a 10 min incubation period. ELISAs were developed and stopped using the same protocol as above. Titers were represented as Area Under the Curve values.

### Statistical analyses

All statistical analyses were performed using GraphPad Prism version 9. Statistical analyses were performed with *n* ≥ 8 for K18-hACE2 mice studies challenged with WA-1, *n* ≥ 3 for K18-hACE2 mice studies challenged with Delta variant, and *n* ≥ 3 for the CD1 mice studies. Error bars represent standard deviation. Ordinary one-way ANOVA with Dunnett’s multiple comparisons test or Two-Way ANOVA with Tukey’s multiple comparisons test were used with single pooled variance for data sets following a normal distribution and Kruskal–Wallis with Dunn’s multiple comparisons test for non-parametric distributed datasets. Kaplan–Meier survival curves were utilized, and Log-rank (Mantel–Cox) test were used to test the significance of survival between sample groups.

### Reporting summary

Further information on research design is available in the Nature Research Reporting Summary linked to this article.

## Supplementary information

Supplementary data 1

Supplementary data 2

Supplementary figures and figure legends pdf

REPORTING SUMMARY

## Data Availability

The datasets generated during and/or analyzed in this study are available from the corresponding author on reasonable request. Raw Illumina RNAseq reads were deposited on SRA at accession number PRJNA797362.
