# Supplementary material for: Intranasal administration of BReC-CoV-2 COVID-19 vaccine protects K18-hACE2 mice against lethal SARS-CoV-2 challenge
Source: NPJ Vaccines. 2022 Mar 14;7:36. doi: 10.1038/s41541-022-00451-7 (PMC8921182; doi:10.1038/s41541-022-00451-7)
Supplement: Supplementary file 3 — Supplementary figures and figure legends pdf [file 41541_2022_451_MOESM3_ESM.pdf]

Supplementary Table 1

Vaccine route	SARS-CoV-2 antigen	Adjuvant
IN	RBD	BECC 438
IN	RBD	BECC 470
IN	RBD-EcoCRM	no adjuvant
IN	RBD-EcoCRM	IRI
IN	RBD-EcoCRM	CpG
IN	RBD-EcoCRM	BECC 438
IN	RBD-EcoCRM	BECC 470
IM	RBD	BECC 438
IM	RBD	BECC 470
IM	RBD-EcoCRM	no adjuvant
IM	RBD-EcoCRM	IRI
IM	RBD-EcoCRM	CpG
IM	RBD-EcoCRM	BECC 438
IM	RBD-EcoCRM	BECC 470

Supplementary Figure 1

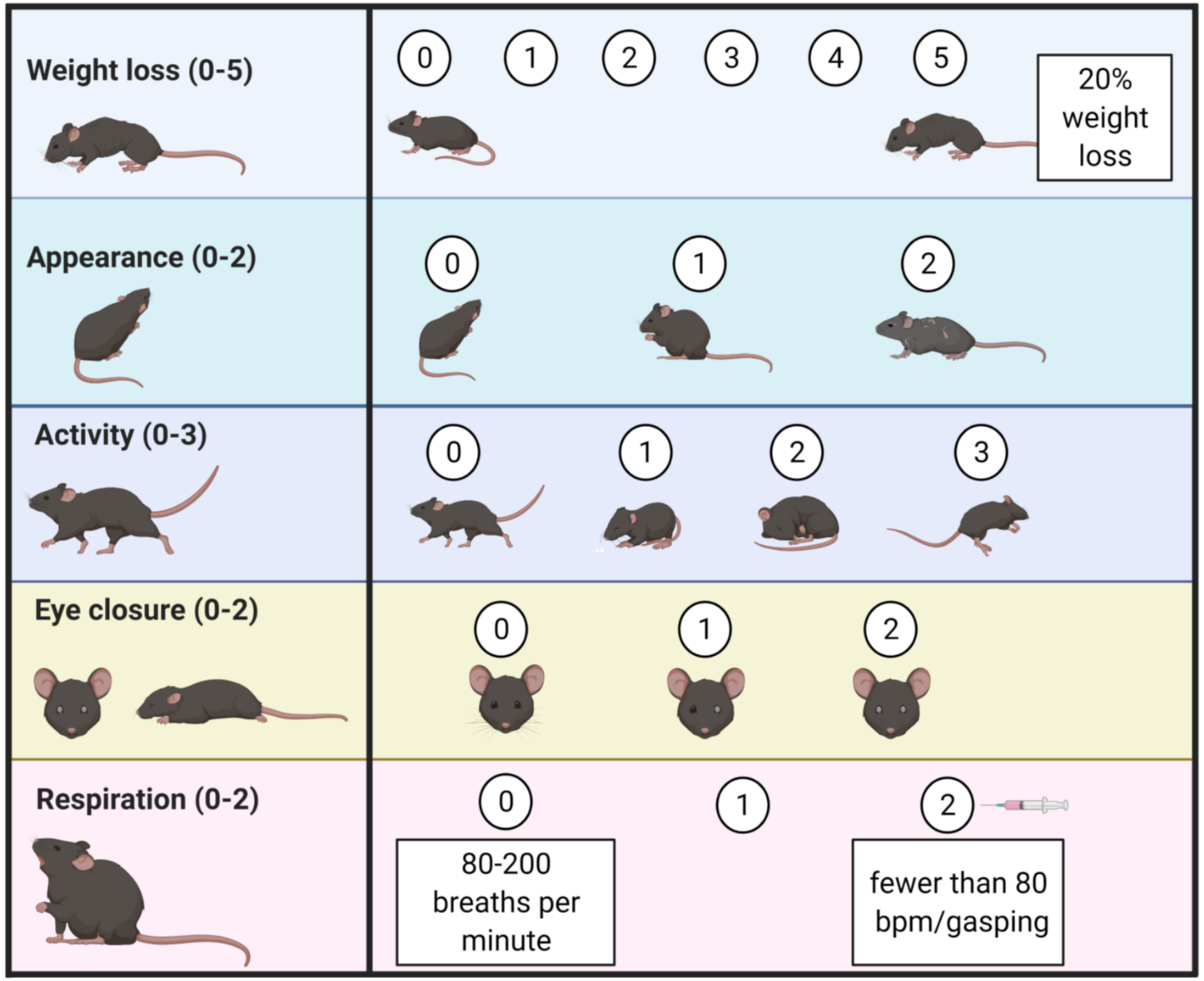

Supplementary Figure 2

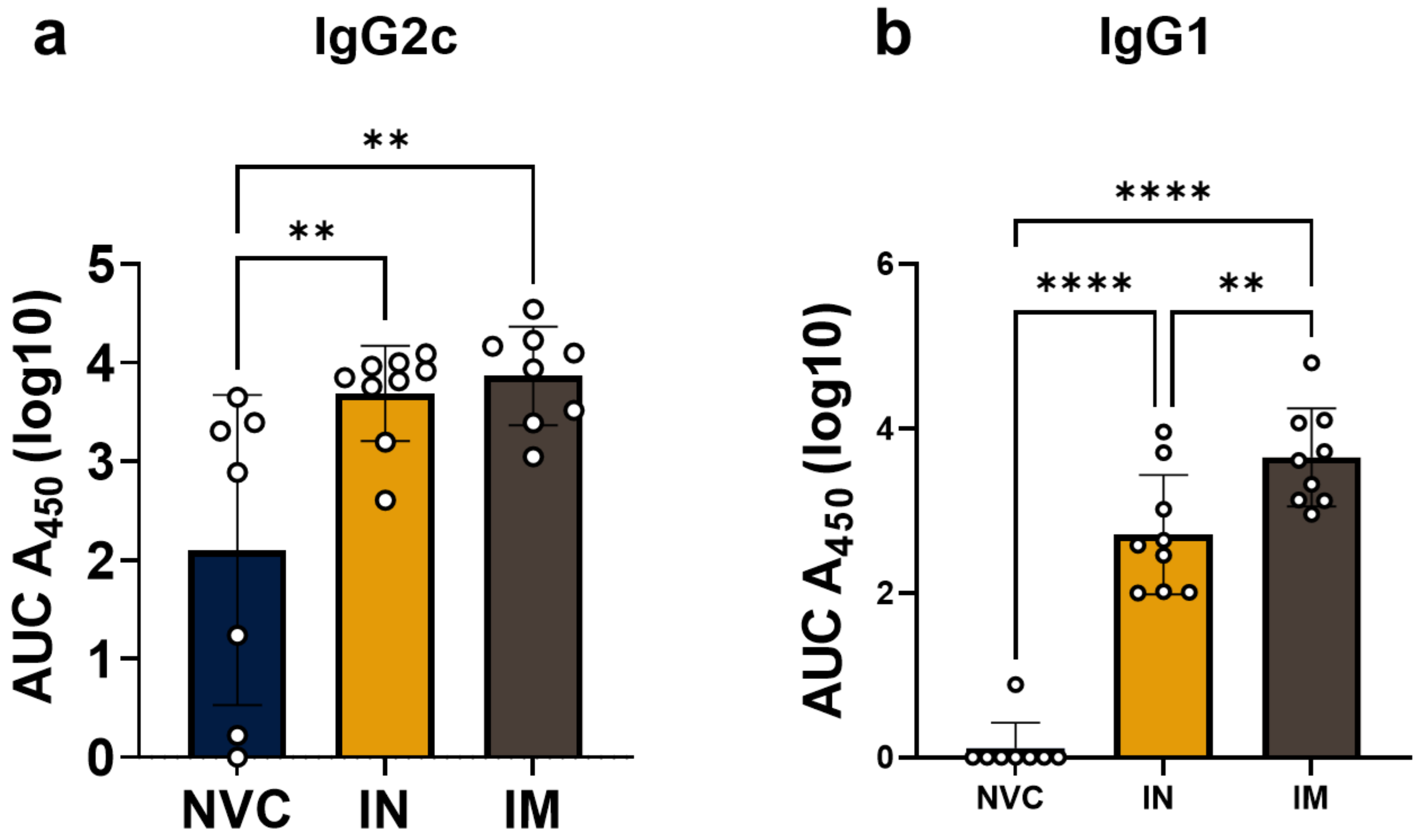

Supplementary Figure 3

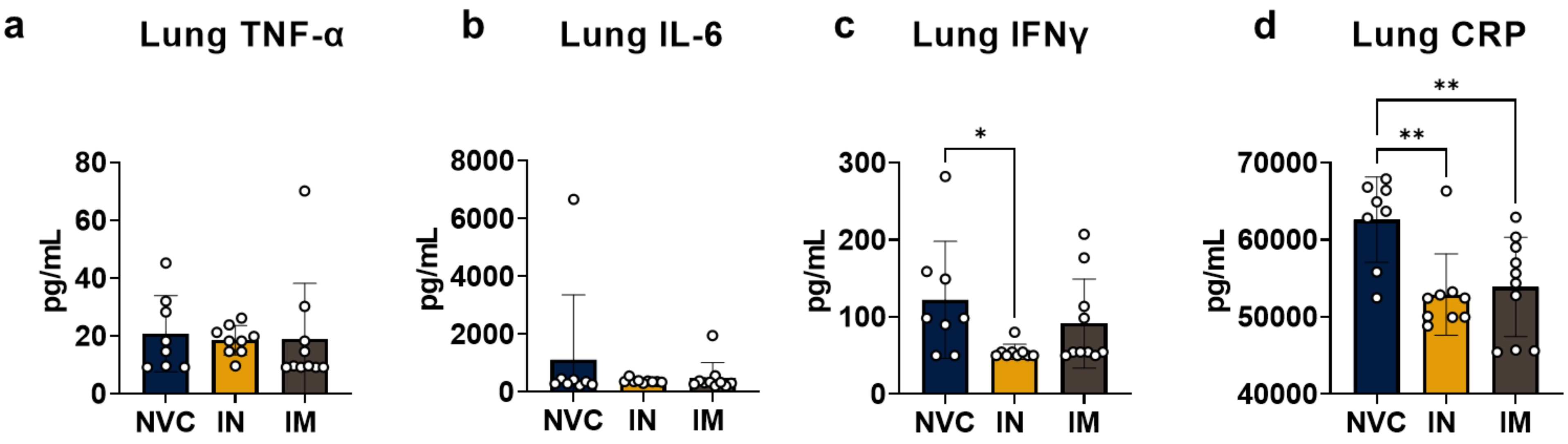

Supplementary Table 1. COVID-19 vaccine formulations and routes for CD1 immunogenicity studies. 7 different COVID-19 vaccine formulations and administered routes used in CD-1 mice immunogenicity studies.

Supplementary Data 1. Statistical analysis of RBD IgG titers in CD1 mice vaccinated with BREC-CoV-2 or RBD + BECC470 (figure 2). Two-way ANOVA with Tukey's multiple comparisons test was performed on the RBD IgG titers represented in AUC values on week 1, 2, 4 5 and 22 weeks.

Supplementary Data 2. RNAseq expression browser (figure 9).

Supplementary Figure 1. Disease scoring schematic. 5 categories of disease manifestations that are observed daily. Symptoms in each category are scored from 0 being no symptoms to the highest number being the worst symptoms. All scores from each category are added up for each mouse, and if a mouse scores a 5 or above, the mouse will be humanely euthanized.

Supplementary Figure 2. BReC-CoV-2 vaccination demonstrated a balanced Th1/Th2 response. A) Serum IgG2c represented by log10 AUC450 in challenged mice. B) Serum IgG1 represented by log10 AUC450 in challenged mice. Results represented as mean ± SD. Ordinary one-way ANOVA with Tukey's multiple comparisons test was performed for statistical analyses. P=0.0047** (IgG2c) and P<0.0001**** and P=0.0063** (IgG1).

Supplementary Figure 3. **IN BReC-CoV-2 decreased IFNγ in the lung.** A) TNF-α (pg/mL) in the lung supernatant. B) IL-6 measured in the lung supernatant. C) IFNγ measured in the lung supernatant. Ordinary one-way ANOVA with Dunnett's multiple comparisons test was used for statistical analysis. P=0.0325*. D) CRP measured in the lung supernatant. Results represented as mean ± SD. Ordinary one-way ANOVA with Dunnett's multiple comparisons test was used for statistical analysis P=0.0041 (NVC vs. IN) and P=0.0078 (NVC vs. IM).
