# Supplementary material for: Intranasal administration of BReC-CoV-2 COVID-19 vaccine protects K18-hACE2 mice against lethal SARS-CoV-2 challenge
Source: NPJ Vaccines. 2022 Mar 14;7:36. doi: 10.1038/s41541-022-00451-7 (PMC8921182; doi:10.1038/s41541-022-00451-7)
Supplement: Supplementary file 4 — REPORTING SUMMARY [file 41541_2022_451_MOESM4_ESM.pdf]

nature portfolio

Corresponding author(s): F. Heath Damron

Last updated by author(s): December 20, 2021

Reporting Summary

Nature Portfolio wishes to improve the reproducibility of the work that we publish. This form provides structure for consistency and transparency in reporting. For further information on Nature Portfolio policies, see our Editorial Policies and the Editorial Policy Checklist.

Statistics

For all statistical analyses, confirm that the following items are present in the figure legend, table legend, main text, or Methods section.

n/a	Confirmed	
☐	☒	The exact sample size (*n*) for each experimental group/condition, given as a discrete number and unit of measurement
☐	☒	A statement on whether measurements were taken from distinct samples or whether the same sample was measured repeatedly
☐	☒	The statistical test(s) used AND whether they are one- or two-sided<br>*Only common tests should be described solely by name; describe more complex techniques in the Methods section.*
☒	☐	A description of all covariates tested
☐	☒	A description of any assumptions or corrections, such as tests of normality and adjustment for multiple comparisons
☐	☒	A full description of the statistical parameters including central tendency (e.g. means) or other basic estimates (e.g. regression coefficient) AND variation (e.g. standard deviation) or associated estimates of uncertainty (e.g. confidence intervals)
☐	☒	For null hypothesis testing, the test statistic (e.g. *F*, *t*, *r*) with confidence intervals, effect sizes, degrees of freedom and *P* value noted<br>*Give P values as exact values whenever suitable.*
☒	☐	For Bayesian analysis, information on the choice of priors and Markov chain Monte Carlo settings
☒	☐	For hierarchical and complex designs, identification of the appropriate level for tests and full reporting of outcomes
☒	☐	Estimates of effect sizes (e.g. Cohen's *d*, Pearson's *r*), indicating how they were calculated

Our web collection on statistics for biologists contains articles on many of the points above.

Software and code

Policy information about availability of computer code

Data collection	ELISA absorbance readings were determined using the Biotek Gen5 software (Synergy H1 plate reader). Cytokines were evaluated using the Luminex MAGPIX. MSD neutralization data was obtained using the MSD QuickPlex SQ120. qPCR viral RNA quantification was performed on StepONEPlus Real-time PCR system (applied biosystems by Thermo Fisher Scientific).
Data analysis	CLC genomics workbench version 21.0.5 was used for read trimming for quality and mapping to the Mus musculus reference genome. Statistical analysis was performed with the Differential Gene Expression tool (CLC genomics workbench) and genes were annotated with the reference mouse gene ontology terms. Genes with an FDR p value of <0.05 were considered differentially regulated. Volcano plot was generated with statistically significant genes. Genes of interest were plotted in a heat map that was generated in GraphPad version 9.0. Genes that were differentially regulated were further analyzed via the online WEB-based GEne SeT AnaLysis Toolkit using over-representation analysis using the mouse enrichment category gene ontology and biological process. Heat maps were generated using Morpheus. Microsoft Excel and Prism Graphpad were used to organize, create figures, and perform statistics. MSD Discovery Workbench 4.0 software was used to determine neutralization electrochemiluminescence values. Luminex xPONENT for MAGPIX version 4.3 was used to assess concentration of cytokines.

For manuscripts utilizing custom algorithms or software that are central to the research but not yet described in published literature, software must be made available to editors and reviewers. We strongly encourage code deposition in a community repository (e.g. GitHub). See the Nature Portfolio guidelines for submitting code & software for further information.

Data

Policy information about availability of data

All manuscripts must include a data availability statement. This statement should provide the following information, where applicable:

- Accession codes, unique identifiers, or web links for publicly available datasets
- A description of any restrictions on data availability
- For clinical datasets or third party data, please ensure that the statement adheres to our policy

Provide your data availability statement here.

Field-specific reporting

Please select the one below that is the best fit for your research. If you are not sure, read the appropriate sections before making your selection.

☒ Life sciences ☐ Behavioural & social sciences ☐ Ecological, evolutionary & environmental sciences

For a reference copy of the document with all sections, see nature.com/documents/nr-reporting-summary-flat.pdf

Life sciences study design

All studies must disclose on these points even when the disclosure is negative.

Sample size	The minimum animal size was n=3 and maximum animal size was n=10.
Data exclusions	No data was excluded form this study
Replication	Mice used in this study were counted as biological replicates.<br>For qPCR analysis for viral RNA, each sample obtained from a mouse was run in triplicate and averaged to obtain copy number.<br>ELISA, cytokine, Histopathology, and MSD neutralization analysis was performed using samples obtained from mice as biological replicates.<br>RNA sequencing analysis utilized n=5 from each experimental group (NVC, IN, and IM).
Randomization	Randomization of samples was not performed.
Blinding	Investigators were not blinded to the group allocation during data collection or analysis. However, technicians disease scoring the animals as well as performing ELISAs, qPCR, and neutralization were blinded to the groups. Pathologist scoring the lungs were blinded to the sample groups.

Reporting for specific materials, systems and methods

We require information from authors about some types of materials, experimental systems and methods used in many studies. Here, indicate whether each material, system or method listed is relevant to your study. If you are not sure if a list item applies to your research, read the appropriate section before selecting a response.

Materials & experimental systems

n/a	Involved in the study	
☐	☒	Antibodies
☐	☒	Eukaryotic cell lines
☒	☐	Palaeontology and archaeology
☐	☒	Animals and other organisms
☒	☐	Human research participants
☒	☐	Clinical data
☒	☐	Dual use research of concern

Methods

n/a	Involved in the study	
☒	☐	ChIP-seq
☒	☐	Flow cytometry
☒	☐	MRI-based neuroimaging

Antibodies

Antibodies used	All secondary antibodies used for serological analysis via ELISA were obtained from Novus Biologicals.<br>Secondary antibodies (Goat anti-mouse) were conjugated to HRP.<br>Mouse IgG: Novus Biologicals NBP1-75130<br>Mouse IgA: Novus Biologicals NB7504<br>Mouse IgG1: Novus Biologicals NB7511<br>Mouse IgG2c: Novus Biologicals NBP2-68519

Validation	*Describe the validation of each primary antibody for the species and application, noting any validation statements on the manufacturer's website, relevant citations, antibody profiles in online databases, or data provided in the manuscript.*

Eukaryotic cell lines

Policy information about cell lines

Cell line source(s)	Both Delta and WA-1 were propagated in Vero E6 Cells obtained from ATCC (ATCC-CRL-1586)
Authentication	Authenticated by ATCC, certificate of analysis available on atcc.org
Mycoplasma contamination	None detected.
Commonly misidentified lines (See ICLAC register)	*Name any commonly misidentified cell lines used in the study and provide a rationale for their use.*

Animals and other organisms

Policy information about studies involving animals; ARRIVE guidelines recommended for reporting animal research

Laboratory animals	Mice used in this study:<br>CD1 IGS outbred<br>Female<br>Charles river<br>strain code: 022<br>Age: 8 weeks old<br>B6.Cg-Tg(K18-ACE2)2Prlmn/J (common name: K18-hACE2)<br>male and female<br>Jackson Laboratory<br>strain #: 034860<br>Age: 10 weeks old
Wild animals	No wild animals were used in study.
Field-collected samples	No field samples were used in study.
Ethics oversight	CD1 outbred mouse immunogenicity studies were performed under the approved West Virginia University IACUC protocol number 2004034204 whereas B6.Cg-Tg(K18-ACE2)2Prlmn/J mouse vaccine and SARS-CoV-2 challenge studies were executed under IACUC protocol number 2009036460. All SARS-CoV-2 challenge studies were conducted in the West Virginia University Biosafety Laboratory Level 3 facility under the IBC protocol number 20-04-01.

Note that full information on the approval of the study protocol must also be provided in the manuscript.
